# Mechanistic studies on covalent assemblies of metal-mediated hemi-aminal ethers†
†Electronic supplementary information (ESI) available. CCDC 1018457. For ESI and crystallographic data in CIF or other electronic format see DOI: 10.1039/c4sc02495h



**DOI:** 10.1039/c4sc02495h

**Published:** 2014-09-10

**Authors:** Hyun Hwa Jo, Ramakrishna Edupuganti, Lei You, Kevin N. Dalby, Eric V. Anslyn

**Affiliations:** a Department of Chemistry , The University of Texas at Austin , Austin , Texas 78712 , USA . Email: anslyn@austin.utexas.edu; b Fujian Institute of Research on the Structure of Matter , Chinese Academy of Sciences , Fuzhou , 350002 , P.R. China . Email: lyou@fjirsm.ac.cn; c Division of Medicinal Chemistry , The University of Texas at Austin , Austin , Texas 78712 , USA . Email: dalby@austin.utexas.edu

## Abstract

The use of reversible covalent bonding in a four-component assembly incorporating chiral alcohols was recently reported to give a method for determining the enantiomeric excess of the alcohols *via* CD spectroscopy. Experiments that probe the mechanism of this assembly, which consists of 2-formylpyridine (**2-PA**), dipicolylamine (**DPA**), Zn(ii) and alcohols to yield zinc complexes of tren-like ligands, are presented. The studies focus upon the mechanism of conversion of a hemi-aminal (**1**) to a hemi-aminal ether (**3**), thereby incorporating the fourth component. It was found that molecular sieves along with 3 to 4 equivalents of alcohol are required to drive the conversion of **1** to **3**. Attempts to isolate an intermediate in this reaction *via* addition of strong Lewis acids led to the discovery of a five-membered ring pyridinium salt (**5**), but upon exposure to Zn(ii) and alcohols gave different products to the assembly. This was interpreted to support the intermediacy of an iminium species. Kinetic studies reveal that the conversion of **1** to **3** is zero-order in alcohol in large excesses of alcohol, supporting rate-determining formation of an intermediate prior to reaction with alcohol. Further, the magnitudes of the rate constants for interconversion of **1** and **3** are similar, supporting the notion that there are similar rate-determining steps (rds) for the forward and reverse reactions. Hammett plots show that the rds involves creation of a negative charge (interpreted as the loss of positive charge), supporting the notion that the decomplexation of Zn(ii) from the assemblies to generate apo-forms of **1** and **3** is rate-determining. The individual mechanistic conclusions are combined to create a qualitative reaction coordinate diagram for the interconversion of **1** and **3**.

## Abstract 2

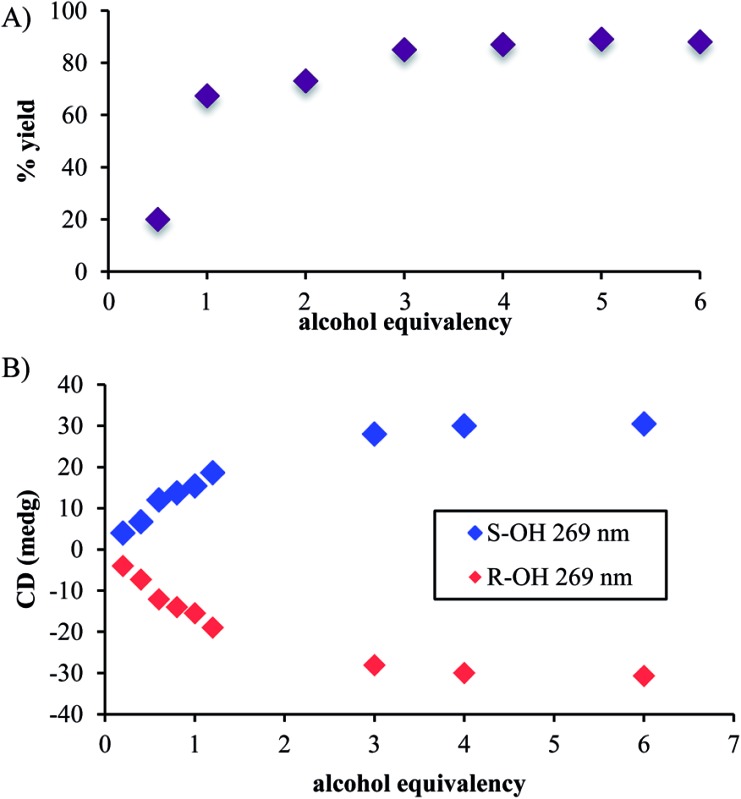
The use of reversible covalent bonding in a four-component assembly incorporating chiral alcohols was recently reported to give a method for determining the enantiomeric excess of the alcohols *via* CD spectroscopy.

## Introduction

Dynamic Covalent Bonding (DCB) can be used to exchange molecular components to reach the thermodynamic minima of a system.^1–6^ In recent decades, DCB has been explored due to applicability in supramolecular chemistry.^7–10^ For example, DCB is useful in creating new molecular receptors, protein ligands and sensors.^11–15^ It is quite common to combine metal-coordination or donor–acceptor interactions with dynamic covalent bonds.^13,16^

One application to which DCB has been applied is the determination of chirality. The discrimination of chiral compounds is essential in the pharmaceutical industry where enantiomeric purity of chiral drugs can greatly influence therapeutic and biological properties.^17^ Much effort has been devoted to creating methods that report the enantiomeric excess (ee) of target chiral building blocks using supramolecular and dynamic covalent bond chemistry.^18–20^

**Scheme 1:**
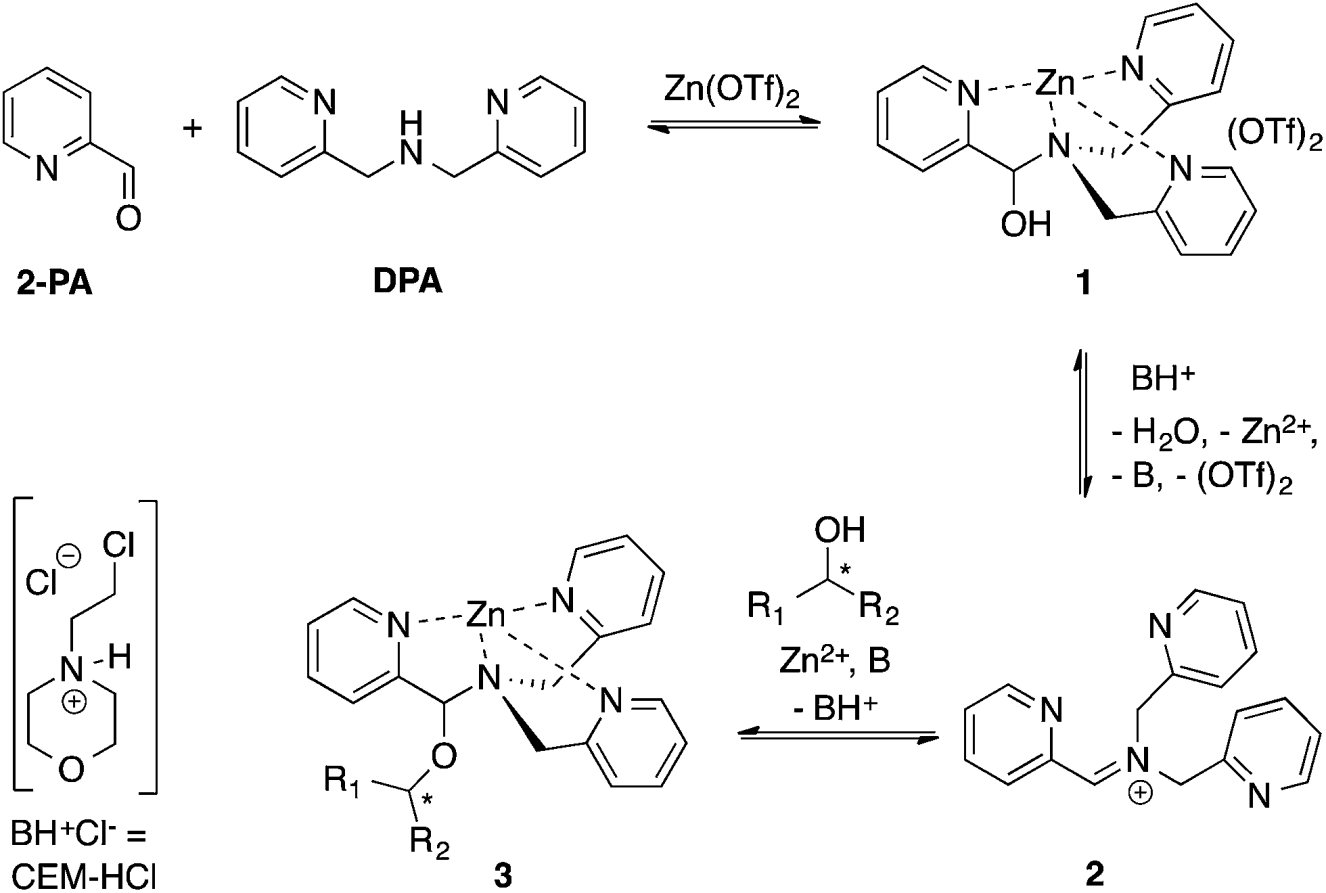
Reversible multicomponent assembly for the binding of chiral secondary alcohols.

Our group reported a one-pot protocol involving multiple dynamic covalent bonds which target chiral alcohols.^21^ This system forms a hemi-aminal (**1**) from three components, which subsequently forms a hemi-aminal ether (**3**) from a fourth component (alcohol) upon dehydration (Scheme 1). The reversibility of the covalent bonds in this assembly enables the exchange of all four components.^22^ The use of this assembly to measure ee values of alcohols has been covered in depth.^21,23,24^ In this paper, a mechanistic investigation of this multi-component assembly is reported. Understanding the mechanism of this assembly should enable further exploitation of dynamic hemi-aminal ether formation in a variety of contexts.^25–27^

## Results and discussion

### I. Extent of alcohol incorporation in the multicomponent assembly

**Scheme 2:**
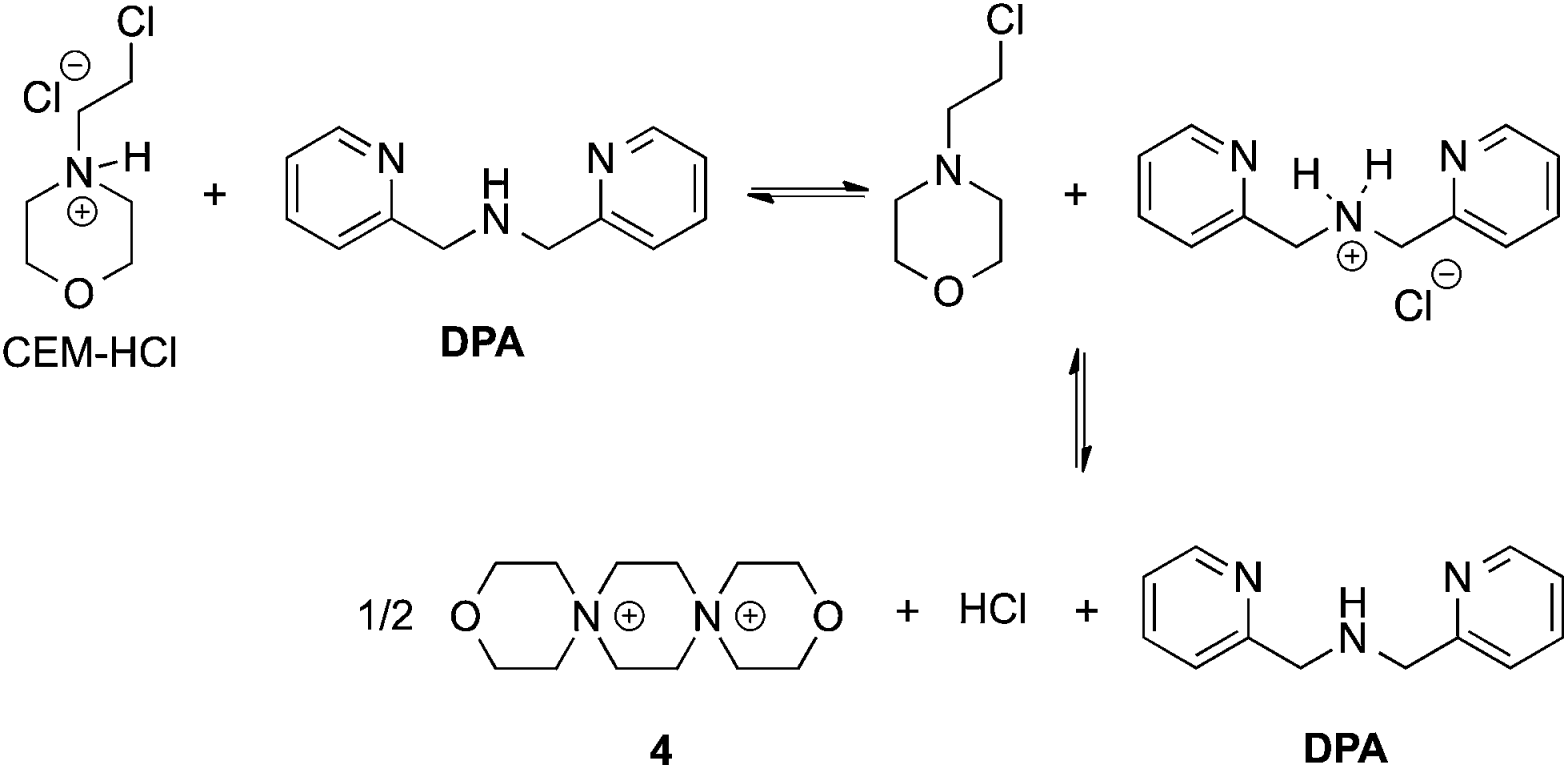
Pathway of CEM–HCl to form 3,12-dioxa-6,9-diazoniadispiro[5.2.5.2]hexadecane, releasing HCl.

In our previous papers describing the use of the four-component assembly given in Scheme 1, we postulated that the reaction proceeded *via* iminium ion **2**, which would then add alcohols to create hemi-aminal ethers that are thermodynamically stabilized *via* binding of Zn(ii) to the tren-like ligand.^21^ Molecular sieves play a major role in the assembly process by scavenging water to drive the equilibrium involving alcohol incorporation. Depending on the absence or presence of molecular sieves in solution, the alcohol incorporation jumps from 40% to 90%, respectively (Table 1). Further, the Brønsted acid (CEM–HCl), used as a catalyst, is critical to the assembly. Without a Brønsted acid, no hemi-aminal ether is formed (Table 1). It was found that CEM–HCl is the most effective acid catalyst, and led to the best yield of the hemi-aminal ether complex when Brønsted acids were screened.^21^ CEM–HCl forms 3,12-dioxa-6,9-diazoniadispiro[5.2.5.2]hexadecane in the presence of **DPA** by slowly releasing hydrochloric acid (Scheme 2).^28^

**Table 1:** Percent yield of hemi-aminal ether complex when number of molecular sieves (3 Å) and presence of Brønsted acid were varied. (Concentration of **2-PA**: 35 mM, **DPA**: 42 mM, alcohol: 175 mM and Zn(ii): 35 mM in acetonitrile)

# of sieves	Brønsted acid	% Alcohol incorporation
0	N	0
0	Y	40
2	N	0
2	Y	88
4	N	0
4	Y	90

**Fig. 1:**
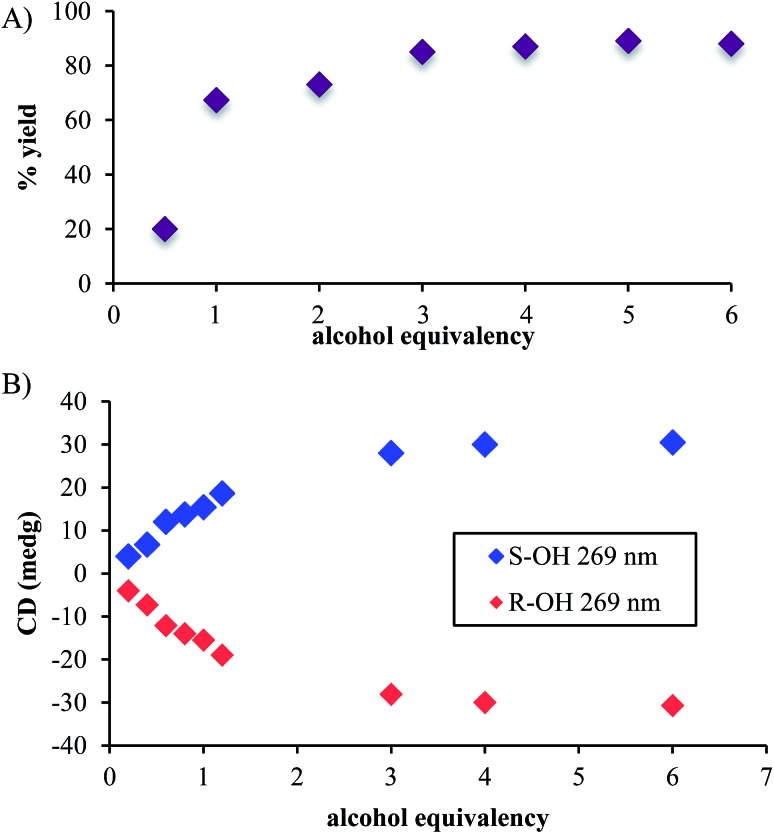
(A) The percent yields and (B) CD intensities of hemi-aminal ether formation when the equivalency of alcohol was varied. (Concentration of **2-PA**: 35 mM, **DPA**: 42 mM and Zn(ii): 35 mM in acetonitrile).

In addition to the necessity of molecular sieves, one needs an excess of alcohol to drive the assembly to completion. Fig. 1A shows the yield of the assembly as a function of the number of equivalents of an alcohol (4-penten-2-ol). As previously reported, the assembly with chiral alcohols results in circular dichroism (CD) signals. Fig. 1B displays the CD intensity as a function of alcohol concentration, which shows that an excess of the alcohol is required to ensure complete assembly. To ensure saturation in the assembly reactions, all experiments for ee determination are conducted using 3 equiv. or more of alcohol.

Because molecular sieves and excess alcohol are required to drive the reaction to completion, it was anticipated that the value of the equilibrium constant (*K*_eq_) between **1** and **3** must be less than 1. The ^1^H NMR chemical shifts of **1** and **3** are distinct, and thus it is a simple matter of integration of the respective resonances to measure a *K*_eq_ value, along with knowledge of the starting concentrations of all reactants: **1**, alcohol and a controlled amount of water (eqn (1)). Thus, with an initial concentration of **1** being 35 mM, water at 35 mM, and alcohol at 175 mM, eqn (1) yielded a *K*_eq_ value of 0.042.1
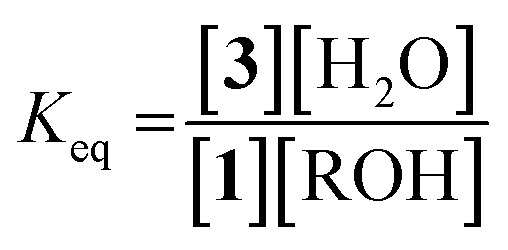



### II. Isolation and characterization of the intermediate

**Scheme 3:**
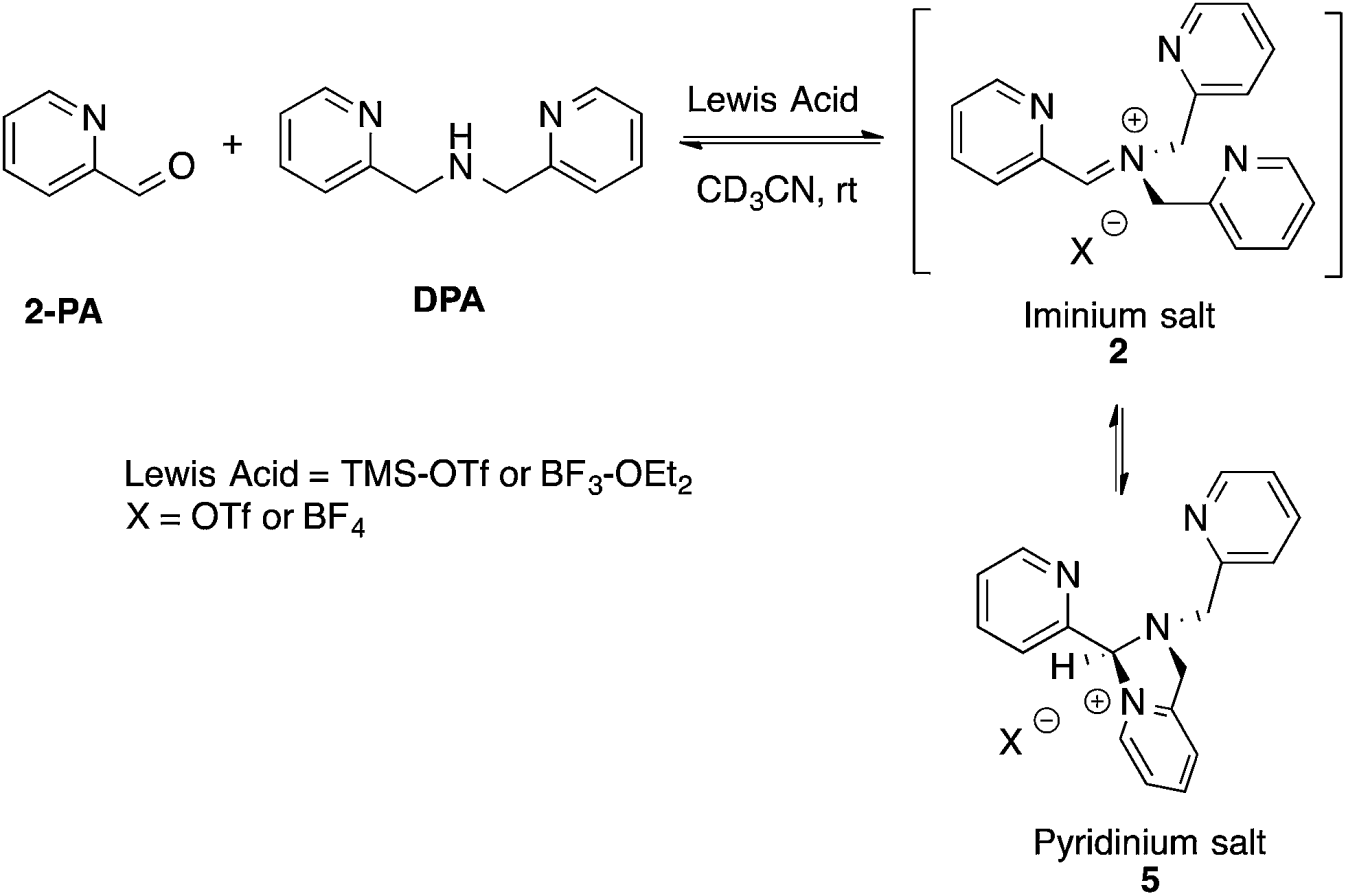
Lewis acid-assisted condensation and formation of a pyridinium salt. The cyclization of **2** to **5** is 5-*endo-trig*, and thus not strictly allowed by Baldwin's rules.

Although not commonly isolated, iminium salts have been characterized previously.^29^ With this precedent in mind, we set out to create iminium **2** as a means to test the validity of its formation as the intermediate in the assembly process shown in Scheme 1. To isolate salt **2**, we used powerful Lewis acids such as TMS–OTf and BF_3_–OEt_2_ to facilitate **DPA** addition to **2-PA** (Scheme 3).

**Fig. 2:**
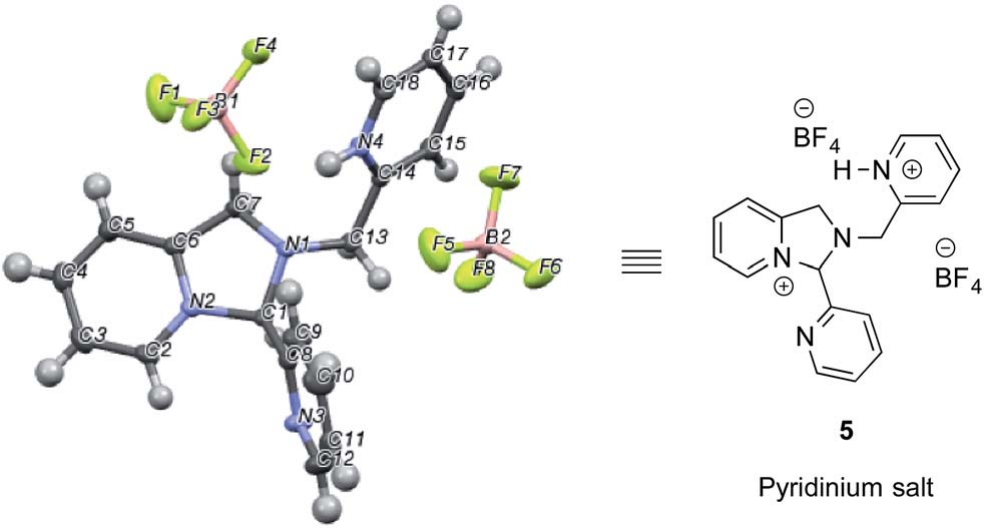
X-ray structure of pyridinium salt **5** created from 2-picolinaldehyde, dipicolylamine and excess BF_3_–OEt_2_.

In an NMR tube, upon addition of one equivalent of TMS–OTf or BF_3_–OEt_2_ to a mixture of **2-PA** and **DPA** in CD_3_CN (60 mM), resonances for a new product along with unreacted **2-PA** were observed. The ^1^H NMR spectrum was not consistent with **2** as the product because two inequivalent CH_2_-groups were formed and the hydrogens on each CH_2_ were diastereotopic (see ESI†). When excess BF_3_–OEt_2_ (more than 2 equiv.) was used to push the addition of **DPA** to completion in acetonitrile, a yellow precipitate was isolated at 0 °C. The precipitate was separated and crystals were grown by slow diffusion of diethyl ether into a solution of the yellow solid in acetonitrile at 0 °C. X-ray diffraction analysis revealed pyridinium salt **5** (Fig. 2).

However, addition of water or alcohol and Zn(OTf)_2_ to the pyridinium salt **5** did not produce good yields of the hemi-aminal **1** or hemi-aminal ether **3**, respectively. Instead, a myriad of additional un-isolable products were created. Therefore, although while pyridinium salt **5** can be isolated, it must not actually be the correct intermediate formed in the assembly. We interpret this evidence as supporting iminium **2** as the true intermediate that reacts with water or an alcohol to create **1** or **3**, respectively. One rationalization for these results come from Baldwin's rules.^30,31^ The cyclization of **2** to **5** is 5-*endo-trig*, which is forbidden by these rules. A second rationalization comes from the expected lifetime of an iminium in the presence of water. For example, in water as the solvent, iminium ions have lifetimes on the order of only picoseconds.^32–35^ Hence, irrespective of the intramolecularity of the pyridine, only in the absence of an external water or alcohol nucleophile does the cyclization occur. Apparently, in the presence of these nucleophiles their intermolecular addition outcompetes the intramolecular addition of pyridine.

### III. Kinetics

**Scheme 4:**
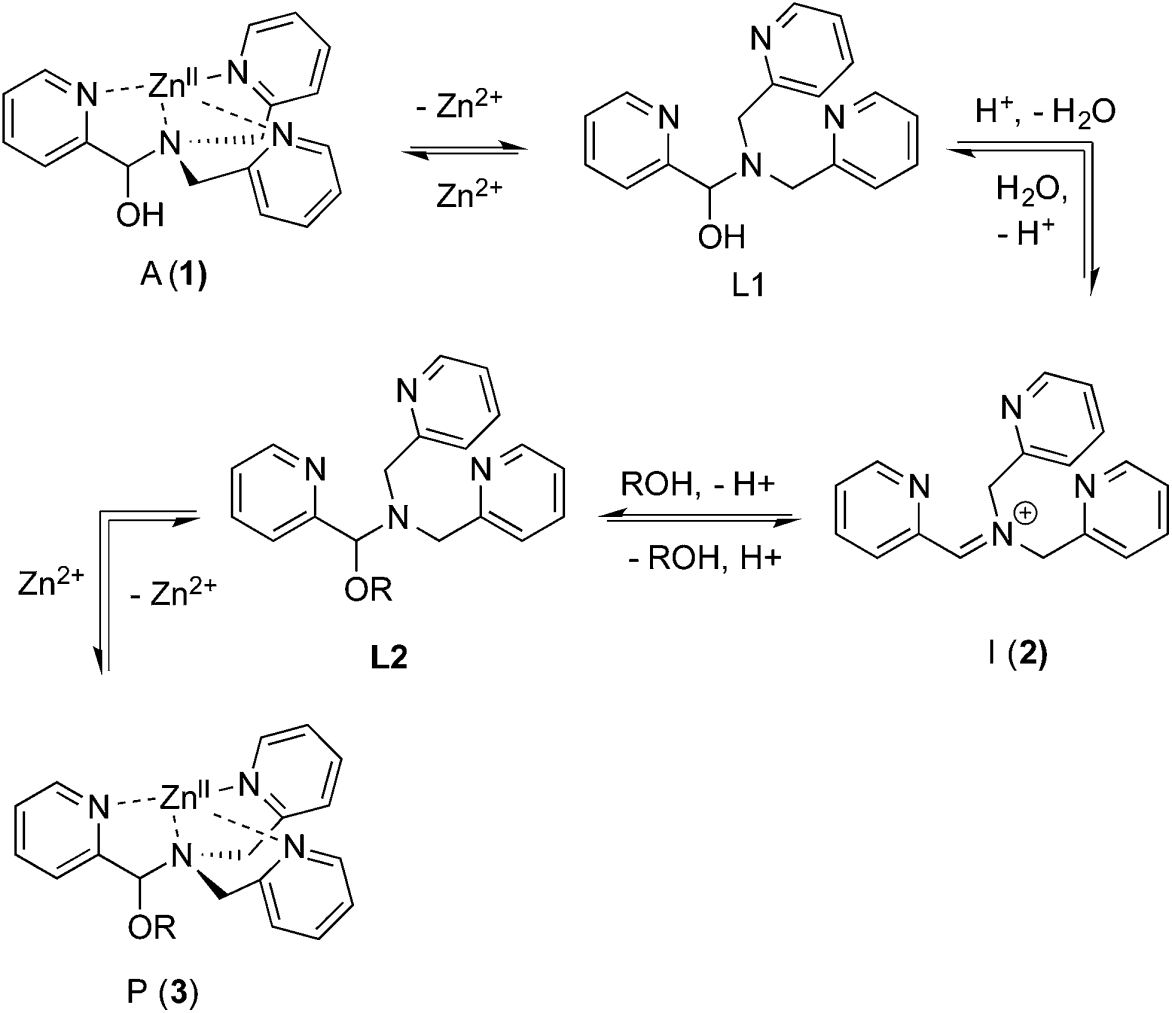
Proposed pathway for interconversion of **1** and **3**.

As described above, the three-component assembly readily forms the hemi-aminal complex **1**, and with assistance of a Brønsted acid catalyst will create the hemi-aminal ether **3** in the presence of an alcohol. Therefore, to explore the mechanism of formation of **3** it was most convenient to start with preformed **1**. A plausible mechanism for the creation of **3** is given in Scheme 4. It starts with loss of Zn(ii) from the tren-like ligand, followed by acid-catalyzed elimination of water to create **2**. Given that **2** is the highest-energy species along the sequence, either of the two steps prior to formation of **2** could be rate-determining. Thus, we set out to determine if loss of metal or elimination of water is the slow step leading to **2**.

**Scheme 5:**
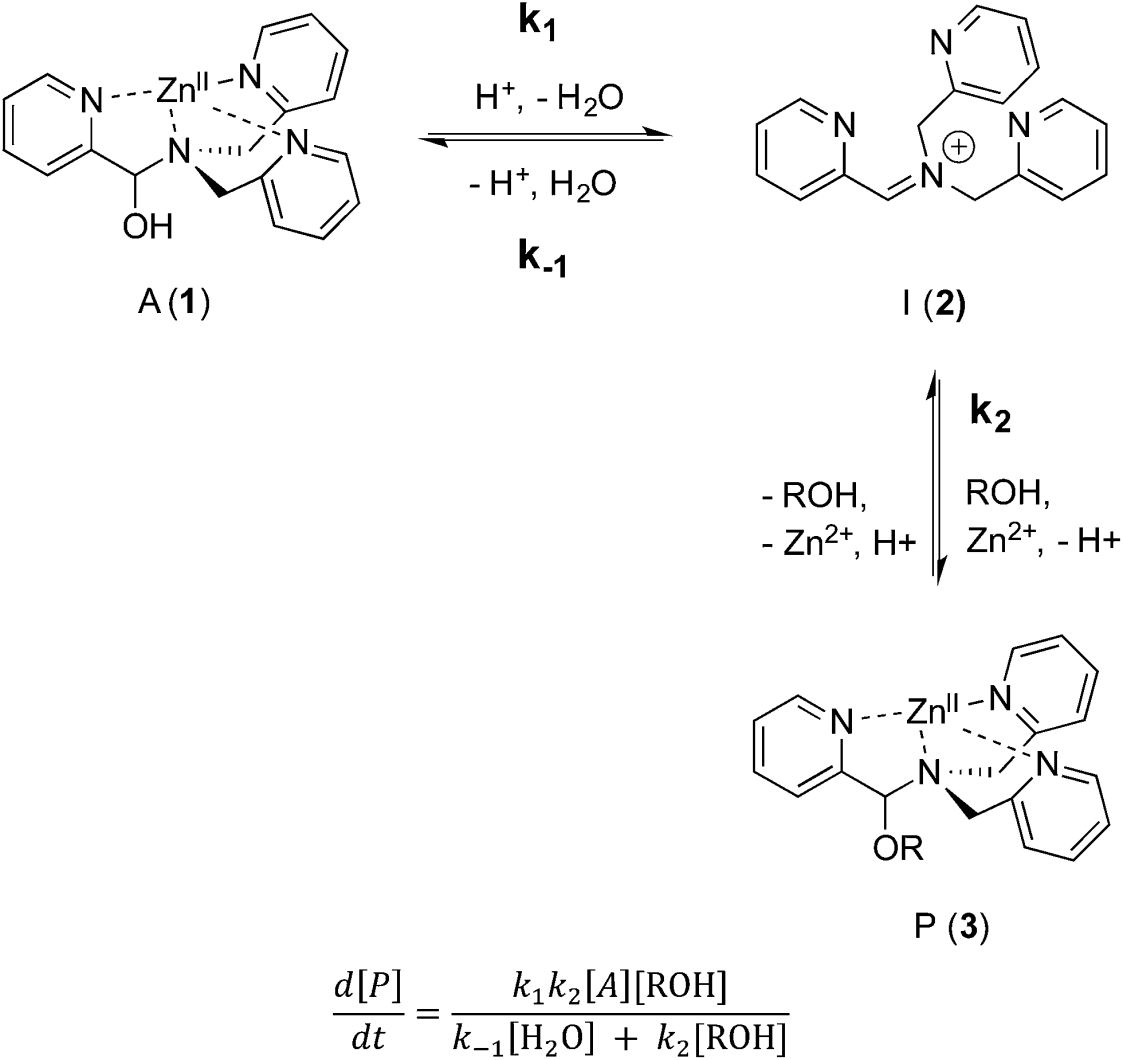
Simplified mechanism and associated rate equation.

**Fig. 3:**
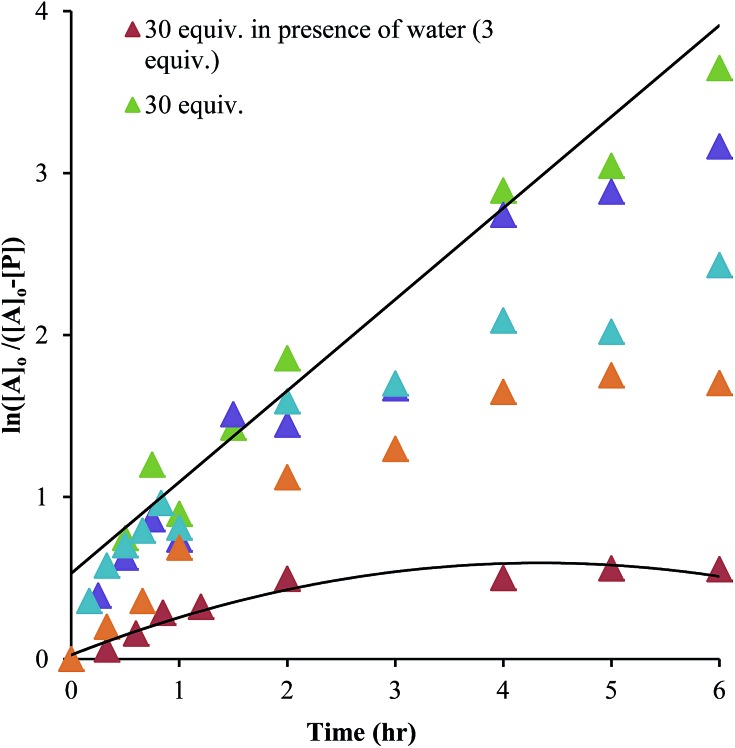
A plot of ln([**A**]_o_/([**A**]_o_ – [**P**]) *versus* time as a function of the equivalents of alcohol. **A** is hemi-aminal **1** and **P** is **3** (all experiments: 35 mM of **2-PA** and Zn(ii) was used for the assembly reaction).

As shown in Scheme 5, the two steps leading to the intermediate can be combined to simplify the mathematical analysis, although either of the two steps could be rate-limiting. The form of the rate expression predicts a second order reaction at low concentrations of alcohol (first order in **1** and alcohol). To test this dependence, we analyzed the reaction with first order in **1** and zero order in alcohol using three equivalents of alcohol (and no molecular sieves). The standard plot of (ln{[**A**]_o_/([**A**]_o_ – [**P**])}) *versus* time gave a plot with significant curvature (Fig. 3), therefore not conforming to first order kinetics. However, the rate expression predicts that the reaction should become increasingly first-order in **1** and zero-order in alcohol as the alcohol concentration increases. The mechanism is analogous to an S_N_1 reaction where at high concentrations of nucleophile a zero order dependence of nucleophile is the norm. As seen in Fig. 3, the kinetic plot becomes increasingly linear, and at 18 or more equivalents of alcohol the plot conforms nicely to pseudo-first order kinetics. Under these conditions the concentration of alcohol is large enough to compete with any residual water or water released during the reaction. The rate constant *k*_–1_ is predicted to be larger than *k*_2_ due to a larger nucleophilicity of water relative to alcohols due to water's smaller size.^36^ Thus, it takes an excess of alcohol to cause the rate expression to simplify to first order in **1** only. When 2 equivalents of water were added at this high concentration of alcohol, the rate drops drastically and the reaction loses first order behaviour, analogous to the common ion effect in an S_N_1 reaction.^37^

**Fig. 4:**
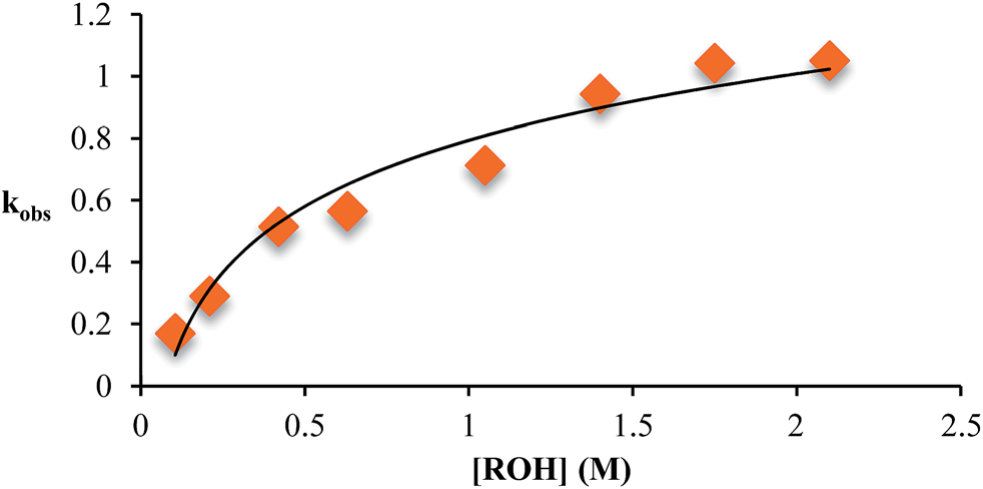
The variation in *k*_obs_ as a function of the starting concentration of alcohol. Concentration of **2-PA** was 35 mM for all experiments.

This kind of kinetics is referred to as saturation kinetics, and it is indicative of a pre-equilibrium prior to the formation of a high energy intermediate which then reacts with the alcohol. Taking the values of the initial slopes in Fig. 3, where the concentration of alcohol was the only variable, leads to the graph shown in Fig. 4. Zero-order dependence on alcohol in the reaction was verified from the plateau in this plot. The experiments given above, however, do not distinguish as to whether the loss of Zn(ii) or the loss of water is the slow step leading to the intermediate.

**Fig. 5:**
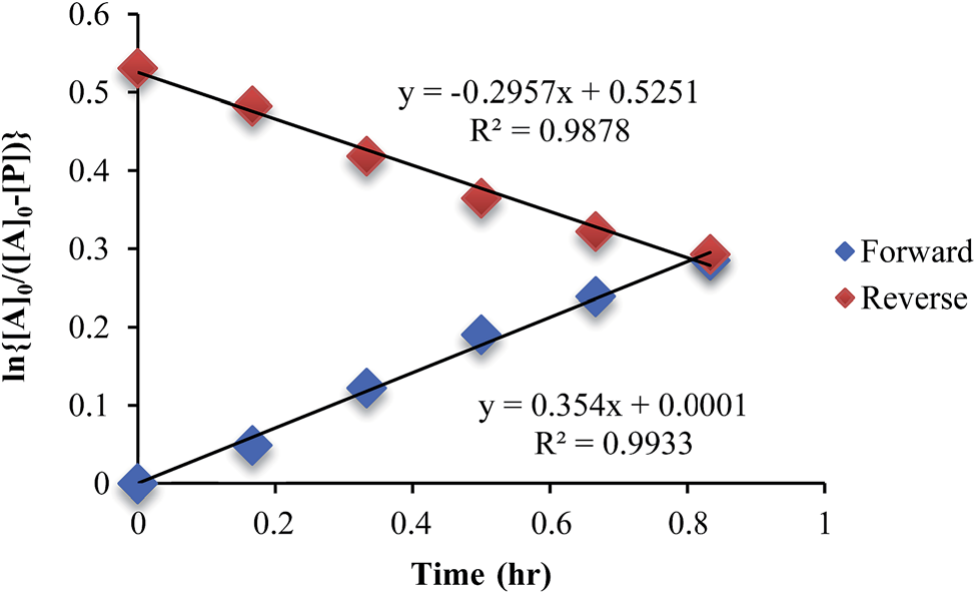
A plot of ln [**A**]_o_/[**A**] *vs.* time in forward and reverse reactions only for the first 10% of the reaction.

The mechanism given in Scheme 4 has analogous steps leading to the intermediate, either starting from reactant **1** or product **3**. The difference is the departure of water or departure of alcohol directly before formation of intermediate **2**. The rate of departure of water is predicted to be slower than that of alcohol due to the increased stability of the hemi-aminal over the hemi-aminal ether, as revealed from the equilibrium constant measured (see above). However, the difference in the rate of loss of Zn(ii) from either the hemi-aminal **1** or the hemi-aminal ether **3** is likely minimal. Thus, to reveal whether metal loss or leaving group departure is the slow step in formation of the intermediate(s), we followed the time course for the forward and reverse reaction during the initial period of the transformations. The reactions involve the addition of alcohol to **1** or water to **3**. All experiments were performed with the concentration of **2-PA** at 0.035 M, and that of alcohol or water fixed at 0.175 M. By fitting a linear line to the first 10% of the reaction, we were able to estimate rate constants of the two reactions (Fig. 5). We find rate constants that are approximately the same (0.30 h^–1^ *vs.* 0.35 h^–1^). This was the first experiment that indicated that the rate-determining step in the hemi-aminal to hemi-aminal ether transformation is loss of the metal.

Although the forward and reverse rate constants are approximately the same, thereby indicating that the rate-determining steps for the forward and reverse reactions are both likely the loss of Zn(ii), it is true that the rate from hemi-aminal ether back to hemi-aminal is slightly faster, as would be predicted because an ROH is anticipated to be a better leaving group. Thus, we sought even stronger evidence that the loss of Zn(ii) is rate-determining, and therefore we performed a Hammett linear free energy analysis.

### IV. Hammett analysis

To further explore the reaction mechanism a Hammett plot was generated. By plotting the log(*k*_x_/*k*_H_) values for various substituted **2-PA**s *versus* the sigma electronic substituent constant (*σ*) a Hammett plot was generated. Hammett plots are informative because they show how reaction mechanisms vary as a function of the electronic changes induced by substituents.^38^

**Fig. 6:**
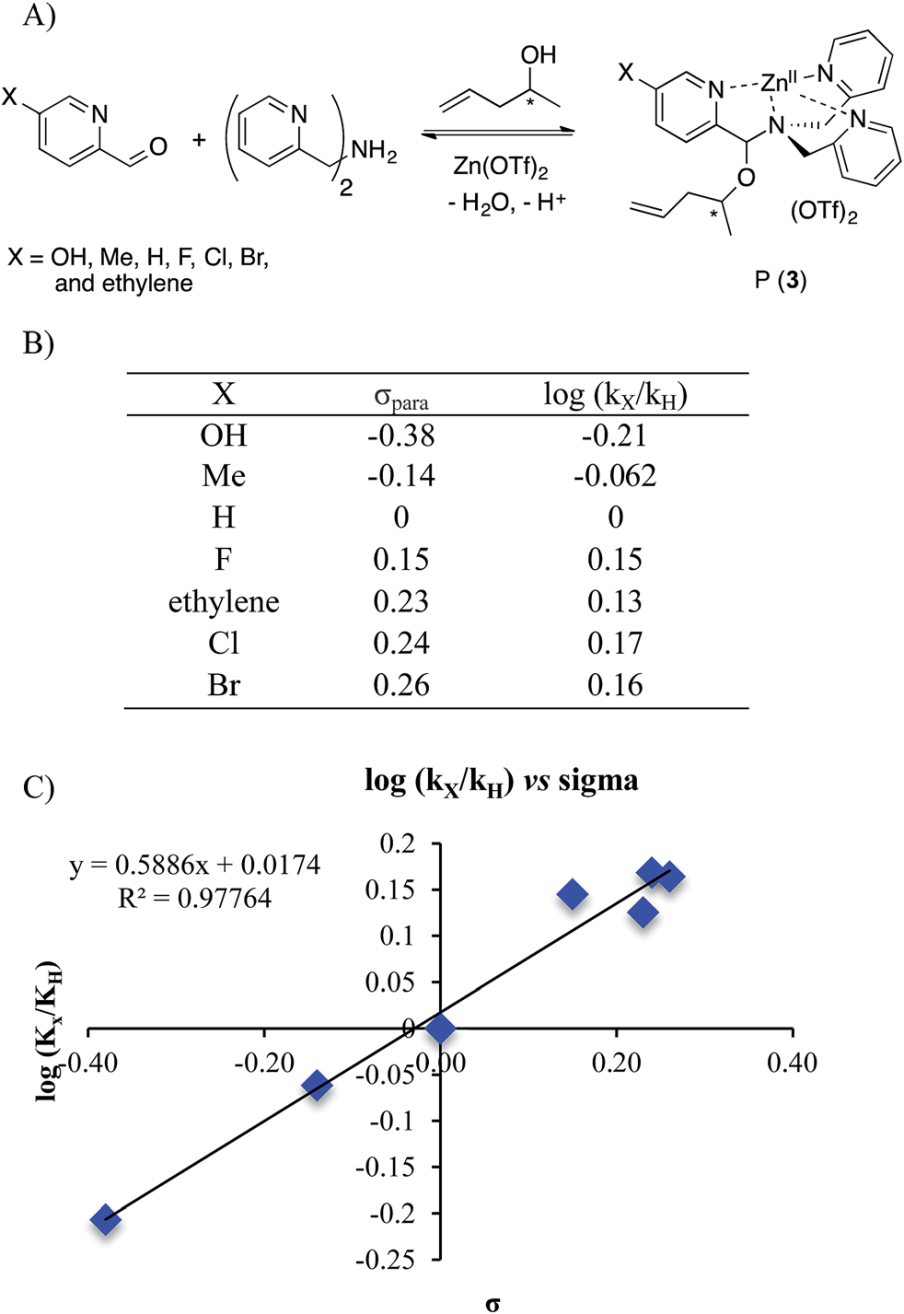
(A) Four-component covalent assembly reactions with various Ar–X structures. (B) *σ_para_* values^38,39^ and corresponding log(*k*_x_/*k*_H_) values for encountered substituents. (C) Hammett plot for the four-component assembly with *para*-substituted 2-picolinaldehyde.

A series of 2-pyridinecarboxaldehyde derivatives bearing electron-donating or electron-withdrawing substituents that are *para* to the aldehyde were investigated for the reaction of **1** to **3** using 4-penten-2-ol as the alcohol (Fig. 6). From the Hammett plot (log(*k*_x_/*k*_H_) *versus σ* ^39^), *ρ* was obtained as the slope. *ρ* describes the sensitivity of the reaction to substituent effects. The calculated *ρ* value from the graph is positive. This leads to the conclusion that negative charge is building during the rds of the assembly, or alternatively that there is a loss of positive charge.

The two possible rate-determining steps for the formation of intermediate **2** either involve loss of the Zn(ii) cation or formation of a positive iminium ion, respectively. Loss of a cation is analogous to increased negative charge, whereas formation of an iminium ion involves generation of a positive charge. Because a positive *ρ* value was found, this supports loss of Zn(ii) as the rds in the conversion of **1** to **3**, and this was also supported by the fact that the forward and reverse reaction rate constants of Scheme 5 are basically the same.

A Hammett plot using *σ* ^+^ was also generated (see ESI†). Such a Hammett plot includes resonance, whereas *σ* primarily reflects induction. The plot using *σ* ^+^ contained significantly more scatter, with a *R* ^2^ value of 0.84 compared to the normal Hammett plot (*R* ^2^ of 0.977). This is in further accordance with our conclusion that the rds is the loss of Zn(ii). If the rds was instead the loss of water, we would predict a better Hammett plot with *σ* ^+^ because **2** is stabilized *via* direct resonance with the substituents. However, the substituents primarily effect metal chelation *via* induction.

### V. Tying it all together

**Fig. 7:**
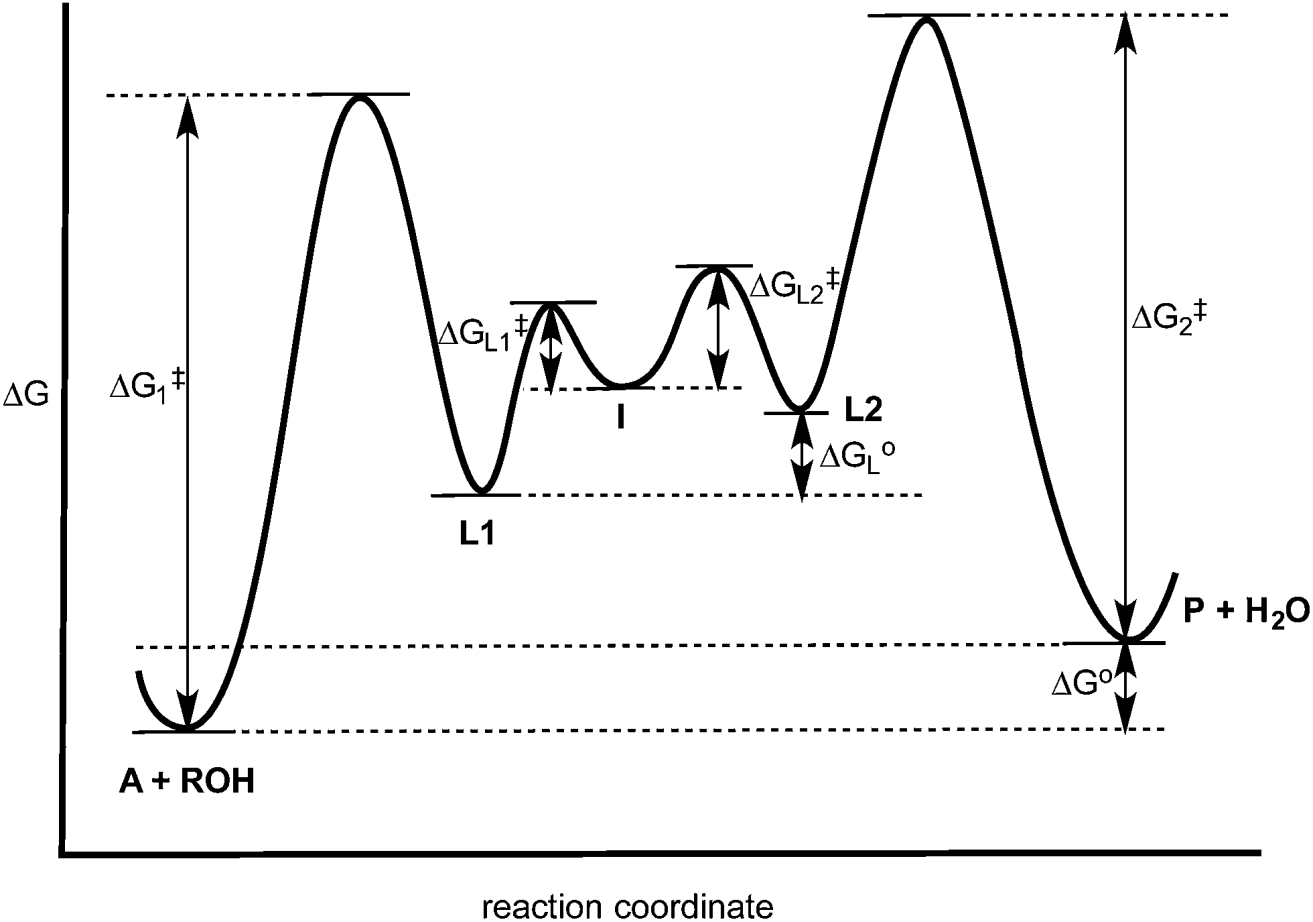
Hypothesized reaction coordinate diagram for the multi-component assembly reaction. See Scheme 4 for the identity of **A**, **L1**, **L2**, **I** and **P**.

The experiments described above allow one to create a qualitative reaction coordinate diagram (Fig. 7) for the interconversion of **1** (**A**) and **3** (**P**). First, because the equilibrium constant for the reaction is less than 1, the energy of **A** and alcohol is placed lower than **P** and water (Δ*G* ^o^). Second, because the loss of Zn(ii) from **A** and **P** was found to have similar rate constants, the barriers leading from **A** and **P** to **L1** and **L2**, respectively, are placed the highest on the diagram and their activation energies are comparable (Δ*G*_1_^‡^ = Δ*G*_2_^‡^). Next, we place intermediate **I** in the center of the diagram, which our results support as being **2** rather than **5**. Third, because water is a better nucleophile than an alcohol, the barrier from **I** to **L1** (Δ*G*_L1_^‡^) is drawn lower than the barrier of **I** to **L2** (Δ*G*_L2_^‡^). The remaining question is the relative energies of **L1** and **L2**, and whether their energy difference is similar to the difference in their activation energies to achieve **I**. However, we postulated above that the bond strengths between OH and OR in **A** and **P** likely do not change significantly whether or not Zn(ii) is bound. Thus, fourth, the energy difference between **L1** and **L2** should be similar to that between **A** and **P**. This reasoning led to the qualitative placement of **L1** and **L2** on the diagram. The third and fourth insights used here to generate the reaction coordinate diagram also led to the conclusion that the activation energy to form **I** from **L1** is higher than from **L2** to **I**. This is consistent with the notion that an alcohol is a better leaving group than water.

## Conclusion

Mechanistic studies of the four-component assembly involving **2-PA**, DPA, Zn(ii), a secondary alcohol, and catalytic acid revealed several insights. First, the equilibrium lies toward hemi-aminal **1**, and thus the creation of a high yield of **3** requires molecular sieves. Further, the reaction can be driven toward **3** *via* the use of excess alcohol. Attempts to isolate the previously-postulated iminium ion **2** instead led to the isolation of a pyridinium salt **5**. Yet, **5** does not give the correct products and its formation is not allowed *via* Baldwin's rules, and thus **5** must form more slowly than the reaction of **2** with water or alcohols. The transformation of **1** to **3** is first order in **1** and zero order in alcohol only at high alcohol concentration, thus showing saturation kinetics in alcohol, analogous to an S_N_1 reaction. This supports the creation of a high-energy intermediate that reacts in a fast step with alcohol. The rate-determining step in the formation of this intermediate is not the acid-catalyzed expulsion of water, but rather the decomplexation of Zn(ii) from the assembly. This conclusion is supported both by the fact that the forward and reverse rate constants for interconversion of **1** and **3** are basically the same, and by a positive Hammett *ρ* value that supports loss of a positive charge in the rds. The mechanistic insights given herein should be informative for other dynamic reactions involving interconversions of hemi-aminals to hemi-aminal ethers.

## References

[cit1] Jin Y., Yu C., Denman R. J., Zhang W. (2013). Chem. Soc. Rev..

[cit2] Gasparini G., Dal Molin M., Lovato A. and Prins L. J., in Supramolecular Chemistry: From Molecules to Nanomaterials, ed. J. W. Steed and P. A. Gale, John Wiley & Sons, Ltd, 2012 10.1002/9780470661345.

[cit3] Miller B. L., Dynamic Combinatorial Chemistry, John Wiley & Sons, 2009.

[cit4] Corbett P. T., Leclaire J., Vial L., West K. R., Wietor J.-L., Sanders J. K. M., Otto S. (2006). Chem. Rev..

[cit5] Lehn J.-M. (2007). Chem. Soc. Rev..

[cit6] Wojtecki R. J., Meador M. A., Rowan S. J. (2011). Nat. Mater..

[cit7] Wilson A., Gasparini G., Matile S. (2014). Chem. Soc. Rev..

[cit8] Jiang X., Lim Y.-K., Zhang B. J., Opsitnick E. A., Baik M.-H., Lee D. (2008). J. Am. Chem. Soc..

[cit9] Yang Y., Pei X.-L., Wang Q.-M. (2013). J. Am. Chem. Soc..

[cit10] Vongvilai P., Ramström O. (2009). J. Am. Chem. Soc..

[cit11] Rowan S. J., Cantrill S. J., Cousins G. R. L., Sanders J. K. M., Stoddart J. F. (2002). Angew. Chem., Int. Ed..

[cit12] Herrmann A. (2014). Chem. Soc. Rev..

[cit13] Lehn J.-M. (2012). Top. Curr. Chem..

[cit14] Black S. P., Sanders J. K. M., Stefankiewicz A. R. (2014). Chem. Soc. Rev..

[cit15] Jin Y., Wang Q., Taynton P., Zhang W. (2014). Acc. Chem. Res..

[cit16] Aricó F. F., Chang T. T., Cantrill S. J. S., Khan S. I. S., Stoddart J. F. J. (2005). Chem.–Eur. J..

[cit17] Hadik P., Szabó L.-P., Nagy E. (2002). Desalination.

[cit18] Reetz M. T., Sell T., Meiswinkel A., Mehler G. (2003). Angew. Chem., Int. Ed..

[cit19] Eelkema R., van Delden R. A., Feringa B. L. (2004). Angew. Chem., Int. Ed..

[cit20] Long J., Hu J., Shen X., Ji B., Ding K. (2002). J. Am. Chem. Soc..

[cit21] You L., Berman J. S., Anslyn E. V. (2011). Nat. Chem..

[cit22] You L., Long S. R., Lynch V. M., Anslyn E. V. (2011). Chem.–Eur. J..

[cit23] You L., Pescitelli G., Anslyn E. V., Di Bari L. (2012). J. Am. Chem. Soc..

[cit24] You L., Berman J. S., Lucksanawichien A., Anslyn E. V. (2012). J. Am. Chem. Soc..

[cit25] Li G., Fronczek F. R., Antilla J. C. (2008). J. Am. Chem. Soc..

[cit26] Star A., Goldberg I., Fuchs B. (2000). Angew. Chem., Int. Ed..

[cit27] Fuchs B., Nelson A., Star A., Stoddart J. F., Vidal S. (2003). Angew. Chem., Int. Ed..

[cit28] Mason J. P., Block H. W. (1940). J. Am. Chem. Soc..

[cit29] Lakhdar S., Tokuyasu T., Mayr H. (2008). Angew. Chem., Int. Ed..

[cit30] Baldwin J. E. (1976). J. Chem. Soc., Chem. Commun..

[cit31] Baldwin J. E., Thomas R. C., Kruse L. I., Silberman L. (1977). J. Org. Chem..

[cit32] Eldin S., Jencks W. P. (1995). J. Am. Chem. Soc..

[cit33] Eldin S., Digits J. A., Huang S.-T., Jencks W. P. (1995). J. Am. Chem. Soc..

[cit34] Eldin S., Jencks W. P. (1995). J. Am. Chem. Soc..

[cit35] Dalby K. N., Jencks W. P. (1997). J. Am. Chem. Soc..

[cit36] Pearson R. G., Sobel H. R., Songstad J. (1968). J. Am. Chem. Soc..

[cit37] Mendham J., Denney R. C., Barnes J. D. and Thomas M. J. K., Vogel's Quantitative Chemical Analysis, Prentice Hall, 6th edn 2000.

[cit38] Ritchie C. D., Sager W. F. (1964). Prog. Phys. Org. Chem..

[cit39] Chen R., Zhang K.-C., Liu L., Li X.-S., Guo Q.-X. (2001). Chem. Phys. Lett..

