# Supplementary material for: Mechanistic studies on covalent assemblies of metal-mediated hemi-aminal ethers
Source: Chem Sci. 2014 Sep 10;6(1):158–64. doi: 10.1039/c4sc02495h (PMC4267293; doi:10.1039/c4sc02495h)
Supplement: Supplementary file 1 [file SC-006-C4SC02495H-s001.pdf]

Electronic Supplementary Material (ESI) for Chemical Science.
This journal is © The Royal Society of Chemistry 2014

Crystallographic Supporting Information for Pyridinium Salt (5).

Mechanistic Studies on Dynamic Multi-Component Covalent Assemblies of Metal-Mediated Hemi-Aminal Ethers†

Hyun Hwa Jo,[a] Ramakrishna Edupuganti,[a,c] Lei You,[b] Kevin N. Dalby[c] and

Eric V. Anslyn*[a]

[a] *Department of Chemistry, The University of Texas at Austin, Austin, Texas, 78712, USA.*

[b] *Fujian Institute of Research on the Structure of Matter, Chinese Academy of Sciences, Fuzhou, 350002, P.R.China.*

[c] *Division of Medicinal Chemistry, The University of Texas at Austin, Austin, Texas, 78712, USA.*

Content

X-ray Experimental Procedures XS1

Figures and Tables XS2

References XS14

X-ray Experimental Procedures:

X-ray Experimental for $(C_{18}H_{18}N_4)^{2+}$ - $2BF_4$: The data crystal was cut from a larger crystal and had approximate dimensions; 0.22 x 0.20 x 0.10 mm. The data were collected at -120 °C on a Nonius Kappa CCD diffractometer using a Bruker AXS Apex II detector and a graphite monochromator with MoK□ radiation (□ = 0.71073Å). Reduced temperatures were maintained by use of an Oxford Cryosystems 600 low-temperature device. A total of 1117 frames of data were collected using □ and ϕ-scans with a scan range of 1.1° and a counting time of 44 seconds per frame. Details of crystal data, data collection and structure refinement are listed in Table 1. Data reduction were performed using SAINT V8.27B.[1] The structure was solved by direct methods using SUPERFLIP[2] and refined by full-matrix least-squares on F^2 with anisotropic displacement parameters for the non-H atoms using SHELXL-2013.[3] Structure analysis was aided by use of the programs PLATON98[4] and WinGX.[5] Most of the hydrogen atoms were calculated in idealized positions. The hydrogen atom bound to N4 was observed in a ΔF map and refined with an isotropic displacement parameter.

The function, $\Sigma w(|F_o|^2 - |F_c|^2)^2$, was minimized, where $w = 1/[(□(F_o))^2 + (0.0508*P)^2 + (2.0034 *P)]$ and $P = (|F_o|^2 + 2|F_c|^2)/3$. $R_w(F^2)$ refined to 0.128, with R(F) equal to 0.0482 and a goodness of fit, S, = 1.01. Definitions used for calculating R(F),$R_w(F^2)$ and the goodness of fit, S, are given below.[6] The data were checked for secondary extinction but no correction was necessary. Neutral atom scattering factors and values used to calculate the linear absorption coefficient are from the International Tables for X-ray Crystallography (1992).[7] All figures were generated using SHELXTL/PC.[8] Tables of positional and thermal parameters, bond lengths and angles, torsion angles and figures are found elsewhere.

Figure 1. Crystal structure of cation of **5**. Displacement ellipsoids are scaled to the 50% probability level.

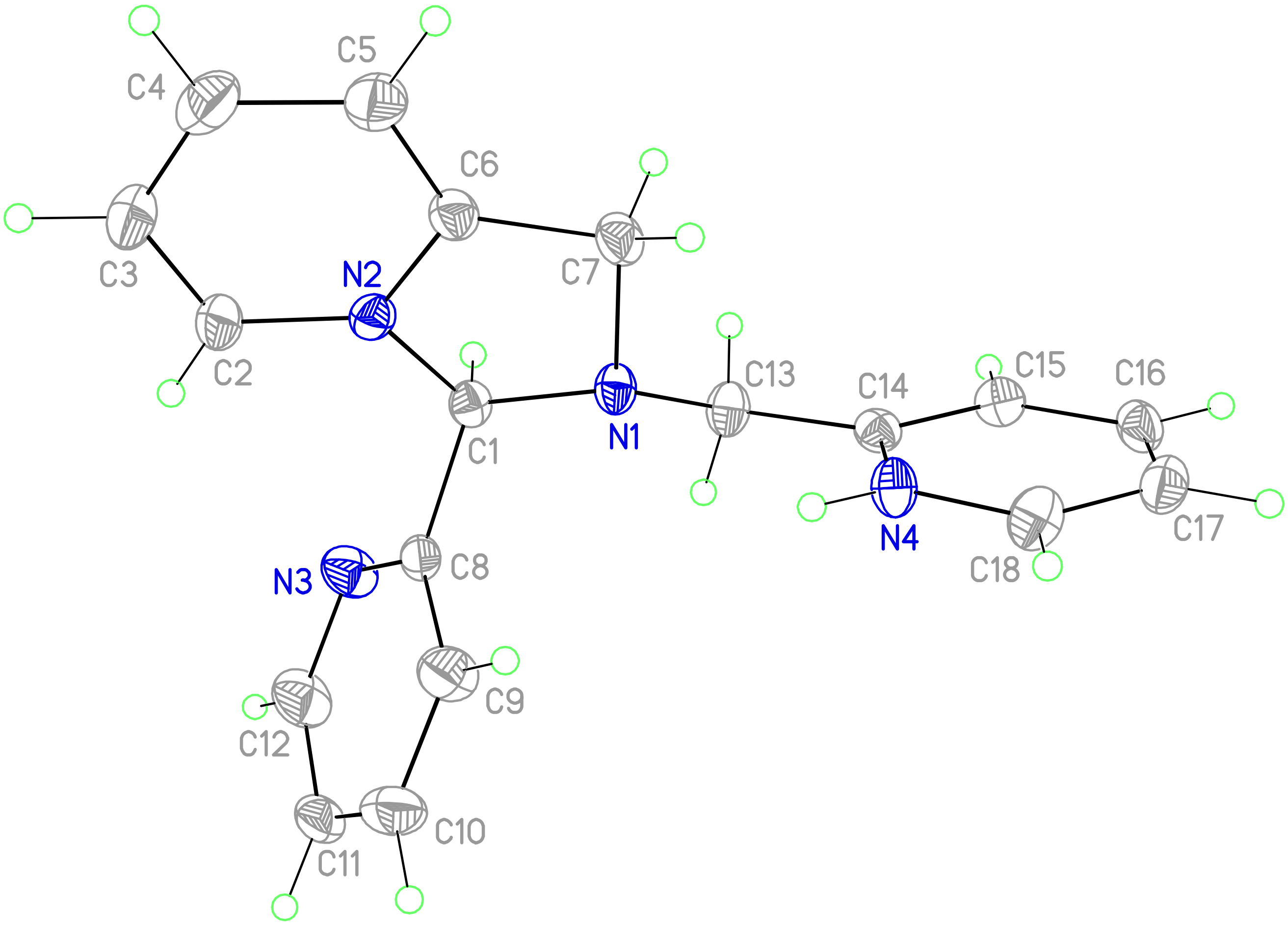

Table 1. Crystal data and structure refinement for pyridinium salt (**5**).

Empirical formula	C18 H18 B2 F8 N4	
Formula weight	463.98	
Temperature	153(2) K	
Wavelength	0.71073 Å	
Crystal system	monoclinic	
Space group	P 21/n	
Unit cell dimensions	a = 7.9704(6) Å	□= 90°.
	b = 9.6387(7) Å	□= 98.556(4)°.
	c = 26.395(2) Å	□ = 90°.
Volume	2005.2(3) $Å^3$	
Z	4	
Density (calculated)	1.537 Mg/m^3	
Absorption coefficient	0.144 mm^{-1}	
F(000)	944	
Crystal size	0.220 x 0.200 x 0.100 mm	
Theta range for data collection	1.560 to 27.499°.	
Index ranges	-10<=h<=10, -12<=k<=12, -34<=l<=34	
Reflections collected	52310	
Independent reflections	4615 [R(int) = 0.0659]	
Completeness to theta = 25.242°	100.0 %	
Absorption correction	Semi-empirical from equivalents	
Max. and min. transmission	1.00 and 0.875	
Refinement method	Full-matrix least-squares on F^2	
Data / restraints / parameters	4615 / 0 / 293	
Goodness-of-fit on F^2	1.012	
Final R indices [I>2sigma(I)]	R1 = 0.0482, wR2 = 0.1119	
R indices (all data)	R1 = 0.0746, wR2 = 0.1281	
Extinction coefficient	n/a	
Largest diff. peak and hole	0.693 and -0.446 e.$Å^{-3}$	

Table 2. Atomic coordinates (x 10^4) and equivalent isotropic displacement parameters ($Å^2$x 10^3) for **5**. U(eq) is defined as one third of the trace of the orthogonalized U^{ij} tensor.

	x	y	z	U(eq)
C1	21(2)	8107(2)	6827(1)	17(1)
C2	-1418(3)	10189(2)	7143(1)	24(1)
C3	-2515(3)	11277(2)	7021(1)	30(1)
C4	-3155(3)	11542(2)	6513(1)	32(1)
C5	-2688(3)	10717(2)	6129(1)	28(1)
C6	-1592(2)	9632(2)	6263(1)	21(1)
C7	-920(3)	8533(2)	5950(1)	22(1)
C8	1508(2)	8213(2)	7251(1)	18(1)
C9	2998(3)	8877(2)	7188(1)	28(1)
C10	4268(3)	8944(3)	7602(1)	35(1)
C11	4012(3)	8351(2)	8058(1)	30(1)
C12	2481(3)	7714(2)	8087(1)	32(1)
C13	880(3)	6504(2)	6214(1)	21(1)
C14	1745(2)	6382(2)	5750(1)	18(1)
C15	1743(2)	5185(2)	5465(1)	21(1)
C16	2592(3)	5154(2)	5046(1)	23(1)
C17	3453(3)	6314(2)	4917(1)	24(1)
C18	3457(3)	7483(2)	5211(1)	24(1)
N1	480(2)	7944(2)	6315(1)	17(1)
N2	-997(2)	9407(2)	6759(1)	18(1)
N3	1226(2)	7631(2)	7689(1)	27(1)
N4	2601(2)	7487(2)	5613(1)	20(1)
B1	2599(3)	11281(2)	5493(1)	26(1)
B2	6047(3)	5942(3)	6481(1)	23(1)
F1	986(2)	11795(2)	5362(1)	63(1)
F2	2668(2)	10282(1)	5877(1)	50(1)
F3	3683(2)	12348(1)	5667(1)	41(1)
F4	3150(2)	10689(2)	5065(1)	45(1)
F5	6127(2)	7336(2)	6603(1)	59(1)
F6	7448(2)	5298(2)	6754(1)	48(1)

F7	6089(2)	5809(2)	5967(1)	56(1)
F8	4593(2)	5380(2)	6614(1)	51(1)

Table 3. Bond lengths [Å] and angles [°] for **5**.

C1-N1	1.461(2)	C12-N3	1.341(3)
C1-N2	1.489(2)	C12-H12	0.95
C1-C8	1.507(3)	C13-N1	1.458(2)
C1-H1	1.00	C13-C14	1.499(3)
C2-N2	1.345(2)	C13-H13A	0.99
C2-C3	1.373(3)	C13-H13B	0.99
C2-H2	0.95	C14-N4	1.343(2)
C3-C4	1.385(3)	C14-C15	1.376(3)
C3-H3	0.95	C15-C16	1.381(3)
C4-C5	1.384(3)	C15-H15	0.95
C4-H4	0.95	C16-C17	1.381(3)
C5-C6	1.375(3)	C16-H16	0.95
C5-H5	0.95	C17-C18	1.369(3)
C6-N2	1.342(2)	C17-H17	0.95
C6-C7	1.491(3)	C18-N4	1.344(3)
C7-N1	1.476(2)	C18-H18	0.95
C7-H7A	0.99	N4-H4N	0.86(3)
C7-H7B	0.99	B1-F1	1.373(3)
C8-N3	1.334(2)	B1-F3	1.378(3)
C8-C9	1.381(3)	B1-F4	1.393(3)
C9-C10	1.378(3)	B1-F2	1.394(3)
C9-H9	0.95	B2-F7	1.368(3)
C10-C11	1.373(3)	B2-F8	1.371(3)
C10-H10	0.95	B2-F5	1.381(3)
C11-C12	1.379(3)	B2-F6	1.382(3
C11-H11	0.95		
N1-C1-N2	100.62(14)	C3-C2-H2	120.9
N1-C1-C8	114.61(15)	C2-C3-C4	119.8(2)
N2-C1-C8	112.80(15)	C2-C3-H3	120.1
N1-C1-H1	109.5	C4-C3-H3	120.1
N2-C1-H1	109.5	C5-C4-C3	120.2(2)
C8-C1-H1	109.5	C5-C4-H4	119.9
N2-C2-C3	118.28(19)	C3-C4-H4	119.9
N2-C2-H2	120.9	C6-C5-C4	118.6(2)

C6-C5-H5	120.7	C14-C15-C16	119.62(18)
C4-C5-H5	120.7	C14-C15-H15	120.2
N2-C6-C5	119.51(18)	C16-C15-H15	120.2
N2-C6-C7	108.76(16)	C17-C16-C15	120.12(18)
C5-C6-C7	131.68(18)	C17-C16-H16	119.9
N1-C7-C6	102.10(15)	C15-C16-H16	119.9
N1-C7-H7A	111.3	C18-C17-C16	119.01(18)
C6-C7-H7A	111.3	C18-C17-H17	120.5
N1-C7-H7B	111.3	C16-C17-H17	120.5
C6-C7-H7B	111.3	N4-C18-C17	119.47(19)
H7A-C7-H7B	109.2	N4-C18-H18	120.3
N3-C8-C9	123.99(18)	C17-C18-H18	120.3
N3-C8-C1	113.52(16)	C13-N1-C1	111.26(14)
C9-C8-C1	122.48(17)	C13-N1-C7	114.30(15)
C10-C9-C8	117.9(2)	C1-N1-C7	107.15(14)
C10-C9-H9	121.0	C6-N2-C2	123.56(17)
C8-C9-H9	121.0	C6-N2-C1	111.24(15)
C11-C10-C9	119.3(2)	C2-N2-C1	124.89(16)
C11-C10-H10	120.4	C8-N3-C12	116.86(19)
C9-C10-H10	120.4	C14-N4-C18	123.22(18)
C10-C11-C12	118.8(2)	C14-N4-H4N	119.2(16)
C10-C11-H11	120.6	C18-N4-H4N	117.6(16)
C12-C11-H11	120.6	F1-B1-F3	109.37(19)
N3-C12-C11	123.1(2)	F1-B1-F4	109.88(19)
N3-C12-H12	118.4	F3-B1-F4	108.60(19)
C11-C12-H12	118.4	F1-B1-F2	111.5(2)
N1-C13-C14	111.31(15)	F3-B1-F2	108.51(19)
N1-C13-H13A	109.4	F4-B1-F2	108.91(18)
C14-C13-H13A	109.4	F7-B2-F8	111.24(19)
N1-C13-H13B	109.4	F7-B2-F5	108.4(2)
C14-C13-H13B	109.4	F8-B2-F5	109.7(2)
H13A-C13-H13B	108.0	F7-B2-F6	109.66(19)
N4-C14-C15	118.55(17)	F8-B2-F6	109.74(19)
N4-C14-C13	118.14(17)	F5-B2-F6	108.08(19)
C15-C14-C13	123.29(17)		

Table 4. Anisotropic displacement parameters ($Å^2$x 10^3) for **5**. The anisotropic displacement factor exponent takes the form: $-2\square^2[h^2 a^{*2}U^{11} + ... + 2 h k a^* b^* U^{12}]$

	U^{11}	U^{22}	U^{33}	U^{23}	U^{13}	U^{12}
C1	18(1)	17(1)	15(1)	1(1)	5(1)	-2(1)
C2	23(1)	30(1)	19(1)	-4(1)	8(1)	0(1)
C3	28(1)	32(1)	31(1)	-7(1)	10(1)	5(1)
C4	26(1)	29(1)	40(1)	0(1)	6(1)	9(1)
C5	28(1)	32(1)	24(1)	3(1)	1(1)	7(1)
C6	21(1)	24(1)	17(1)	0(1)	3(1)	-1(1)
C7	27(1)	24(1)	15(1)	0(1)	1(1)	5(1)
C8	19(1)	18(1)	16(1)	-2(1)	3(1)	2(1)
C9	26(1)	36(1)	23(1)	1(1)	5(1)	-9(1)
C10	22(1)	48(1)	35(1)	-5(1)	2(1)	-9(1)
C11	26(1)	34(1)	26(1)	-10(1)	-9(1)	9(1)
C12	38(1)	40(1)	18(1)	4(1)	-1(1)	0(1)
C13	26(1)	18(1)	20(1)	1(1)	8(1)	1(1)
C14	18(1)	17(1)	16(1)	2(1)	0(1)	2(1)
C15	22(1)	17(1)	24(1)	1(1)	3(1)	-1(1)
C16	27(1)	21(1)	20(1)	-6(1)	0(1)	5(1)
C17	25(1)	28(1)	20(1)	3(1)	7(1)	7(1)
C18	28(1)	22(1)	26(1)	5(1)	10(1)	1(1)
N1	22(1)	17(1)	14(1)	1(1)	5(1)	2(1)
N2	16(1)	21(1)	17(1)	0(1)	4(1)	0(1)
N3	28(1)	34(1)	17(1)	4(1)	2(1)	-5(1)
N4	27(1)	15(1)	20(1)	-1(1)	7(1)	0(1)
B1	32(1)	20(1)	25(1)	-2(1)	5(1)	0(1)
B2	20(1)	30(1)	19(1)	0(1)	1(1)	-3(1)
F1	33(1)	57(1)	98(1)	-20(1)	5(1)	7(1)
F2	103(1)	20(1)	34(1)	-1(1)	28(1)	-14(1)
F3	47(1)	28(1)	46(1)	9(1)	-3(1)	-12(1)
F4	66(1)	42(1)	27(1)	0(1)	9(1)	17(1)
F5	50(1)	42(1)	88(1)	-21(1)	24(1)	-5(1)
F6	26(1)	69(1)	48(1)	25(1)	-1(1)	7(1)

F7	64(1)	83(1)	20(1)	-7(1)	5(1)	1(1)
F8	25(1)	72(1)	56(1)	17(1)	3(1)	-15(1)

Table 5. Hydrogen coordinates (x 10^4) and isotropic displacement parameters ($Å^2$x 10^3) for **5**.

	x	y	z	U(eq)
H1	-725	7319	6900	20
H2	-968	9993	7489	28
H3	-2834	11846	7285	36
H4	-3918	12293	6429	38
H5	-3115	10897	5779	34
H7A	-1795	7825	5835	26
H7B	-500	8932	5646	26
H9	3142	9276	6868	34
H10	5309	9396	7574	42
H11	4875	8380	8348	36
H12	2306	7315	8404	39
H13A	-180	5952	6163	25
H13B	1626	6120	6515	25
H15	1160	4385	5557	25
H16	2584	4334	4846	28
H17	4034	6301	4627	29
H18	4060	8286	5132	29
H4N	2590(30)	8250(30)	5786(9)	30(6)

Table 6. Torsion angles [°] for **5**.

N2-C2-C3-C4	0.0(3)	C14-C13-N1-C7	72.2(2)
C2-C3-C4-C5	-0.2(3)	N2-C1-N1-C13	-156.86(15)
C3-C4-C5-C6	0.5(3)	C8-C1-N1-C13	81.84(19)
C4-C5-C6-N2	-0.5(3)	N2-C1-N1-C7	-31.26(18)
C4-C5-C6-C7	176.7(2)	C8-C1-N1-C7	-152.56(16)
N2-C6-C7-N1	-15.9(2)	C6-C7-N1-C13	153.52(15)
C5-C6-C7-N1	166.7(2)	C6-C7-N1-C1	29.77(19)
N1-C1-C8-N3	-145.71(17)	C5-C6-N2-C2	0.3(3)
N2-C1-C8-N3	99.94(19)	C7-C6-N2-C2	-177.49(18)
N1-C1-C8-C9	35.5(3)	C5-C6-N2-C1	174.15(18)
N2-C1-C8-C9	-78.8(2)	C7-C6-N2-C1	-3.6(2)
N3-C8-C9-C10	-0.1(3)	C3-C2-N2-C6	0.0(3)
C1-C8-C9-C10	178.5(2)	C3-C2-N2-C1	-173.06(18)
C8-C9-C10-C11	0.2(4)	N1-C1-N2-C6	21.66(19)
C9-C10-C11-C12	-0.5(4)	C8-C1-N2-C6	144.23(16)
C10-C11-C12-N3	0.7(4)	N1-C1-N2-C2	-164.59(17)
N1-C13-C14-N4	25.0(2)	C8-C1-N2-C2	-42.0(2)
N1-C13-C14-C15	-156.79(18)	C9-C8-N3-C12	0.3(3)
N4-C14-C15-C16	-0.9(3)	C1-C8-N3-C12	-178.50(18)
C13-C14-C15-C16	-179.09(18)	C11-C12-N3-C8	-0.6(3)
C14-C15-C16-C17	0.7(3)	C15-C14-N4-C18	0.1(3)
C15-C16-C17-C18	0.4(3)	C13-C14-N4-C18	178.36(18)
C16-C17-C18-N4	-1.2(3)	C17-C18-N4-C14	1.0(3)
C14-C13-N1-C1	-166.27(15)		

Table 7. Hydrogen bonds for **5** [Å] and [°].

D-H...A	d(D-H)	d(H...A)	d(D...A)	<(DHA)
C1-H1...F5#1	1.00	2.51	3.160(2)	122
C1-H1...F6#1	1.00	2.43	3.384(2)	160
C2-H2...F6#2	0.95	2.49	3.144(2)	126
C2-H2...F8#2	0.95	2.48	3.391(3)	160
C7-H7A...F7#1	0.99	2.63	3.551(3)	155
C13-H13B...F8	0.99	2.45	3.181(3)	131
C15-H15...F1#3	0.95	2.55	3.327(3)	139
C17-H17...F3#4	0.95	2.46	3.207(2)	136
C17-H17...F7#5	0.95	2.56	3.165(3)	122
C18-H18...F4	0.95	2.43	3.119(3)	130
C18-H18...F4#4	0.95	2.56	3.396(3)	148
N4-H4N...F2	0.86(3)	1.98(3)	2.782(2)	155(2)

Symmetry transformations used to generate equivalent atoms:

#1 x-1,y,z #2 -x+1/2,y+1/2,-z+3/2 #3 x,y-1,z

#4 -x+1,-y+2,-z+1 #5 -x+1,-y+1,-z+1

References:

1) SAINT V8.27B Bruker AXS Inc, (2012), Madison, WI.

2) Palatinus, L. and Chapuis, G. (2007). Superflip. J. Appl. Cryst. 40, 786-790.

3) Sheldrick, G. M. (2008). SHELXL-2013. Program for the Refinement of Crystal Structures. Acta Cryst., A64, 112-122.

4) Spek, A. L. (1998). PLATON, A Multipurpose Crystallographic Tool. Utrecht University, The Netherlands.

5) WinGX 1.64. (1999). An Integrated System of Windows Programs for the Solution, Refinement and Analysis of Single Crystal X-ray Diffraction Data. Farrugia, L. J. J. Appl. Cryst. 32. 837-838.

6) $R_W(F^2) = \{□w(|F_o|^2 - |F_c|^2)^2/□w(|F_o|)^4\}^{1/2}$ where w is the weight given each reflection.
$R(F) = □(|F_o| - |F_c|)/□|F_o|\}$ for reflections with $F_o > 4(□(F_o))$.
$S = [□w(|F_o|^2 - |F_c|^2)^2/(n - p)]^{1/2}$, where n is the number of reflections and p is the number of refined parameters.

7) International Tables for X-ray Crystallography (1992). Vol. C, Tables 4.2.6.8 and 6.1.1.4, A. J. C. Wilson, editor, Boston: Kluwer Academic Press.

8) Sheldrick, G. M. (1994). SHELXTL/PC (Version 5.03). Siemens Analytical X-ray Instruments, Inc., Madison, Wisconsin, USA.
