# Supplementary material for: Mechanistic studies on covalent assemblies of metal-mediated hemi-aminal ethers
Source: Chem Sci. 2014 Sep 10;6(1):158–64. doi: 10.1039/c4sc02495h (PMC4267293; doi:10.1039/c4sc02495h)
Supplement: Supplementary file 2 [file SC-006-C4SC02495H-s002.pdf]

Electronic Supplementary Material (ESI) for Chemical Science.
This journal is © The Royal Society of Chemistry 2014

Supporting Information

Mechanistic Studies on Dynamic Multi-Component Covalent Assemblies of Metal-Mediated Hemi-Aminal Ethers†

Hyun Hwa Jo,[a] Ramakrishna Edupuganti,[a,c] Lei You,[b] Kevin N. Dalby[c] and Eric V. Anslyn*[a]

[a] *Department of Chemistry, The University of Texas at Austin, Austin, Texas, 78712, USA.*

[b] *Fujian Institute of Research on the Structure of Matter, Chinese Academy of Sciences, Fuzhou, 350002, P.R.China.*

[c] *Division of Medicinal Chemistry, The University of Texas at Austin, Austin, Texas, 78712, USA.*

<u>Content</u>

General Information S1
Experimental Procedures S2
General Procedures for Multi-component Assembly
Synthesis of Pyridinium Salt (**5**)
Hammett Plot using σ^+ S3
NMR and Mass Spectra S4

General Information:

NMR spectra were recorded on Agilent MR 400 at The University of Texas at Austin NMR facility. ESI-mass spectra were obtained on Agilent 6100 at The University of Texas at Austin mass spectrometry facility. Circular dichroism (CD) spectra were recorded on a Jasco J-815 spectropolarimeter at The University of Texas facility.

Experimental Procedures:

General Procedures for Multi-component Assembly

All assembly reactions for kinetics and LEFR studies were performed *in situ* in acetonitrile without isolation and purification. Pyridine-2-carboxyaldehyde (**2-PA**, 35 mM, 1 equiv.), zinc triflate ($Zn(OTf)_2$, 35 mM, 1 equiv.), di-(2-picolyl)amine (**DPA**, 42mM, 1.2 equiv.), 4-penten-2-ol (ROH, 175 mM, 5 equiv. except for the alcohol dependence studies), and 4-(2-chloroethyl)morpholine hydrochloride (CEM-HCl, 35 mM, 1 equiv.) were stirred together in acetonitrile in the presence of 3Å activated molecular sieves. The mixture was stirred at room temperature.

Synthesis of Pyridinium Salt (5)

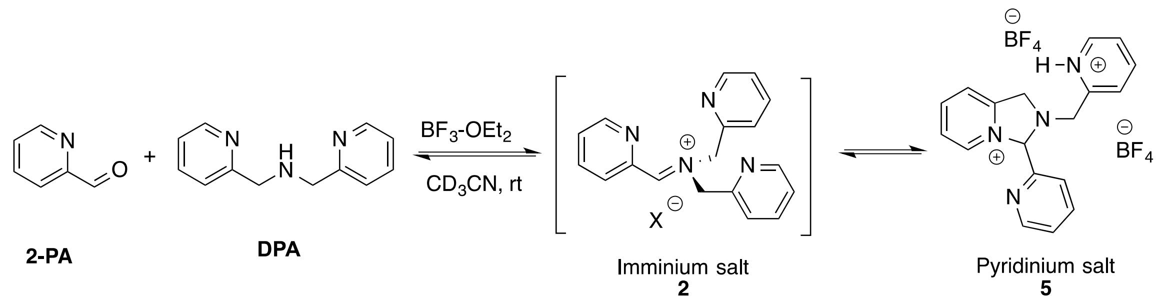

To pyridine-2-carboxaldehyde (**2-PA**, 3.21 mg, 0.03 mmol) in dry CD_3CN solution (60 mM), dipicolylamine (**DPA**, 7.17 mg, 0.036 mmol) was added. Then, BF_3-OEt_2 (5.11 μL, 0.036 mmol) was added dropwise. The reaction mixture was shaken for 3–5 min and then ^1H NMR, ^{13}C NMR, and mass spectrum were recorded.

Hammett Plot using σ^+:

X	K_{eq}	σ^+	log (k_X/k_H)
OH	16.9	-0.92	-0.21
Me	23.6	-0.31	-0.062
H	27.2	0	0
F	38.0	-0.07	0.14
alkyne	36.3	-	0.13
Cl	40.1	0.11	0.17
Br	39.7	0.15	0.16

Table S2. σ^+ values and corresponding log (k_X/k_H) values for encountered substituents.

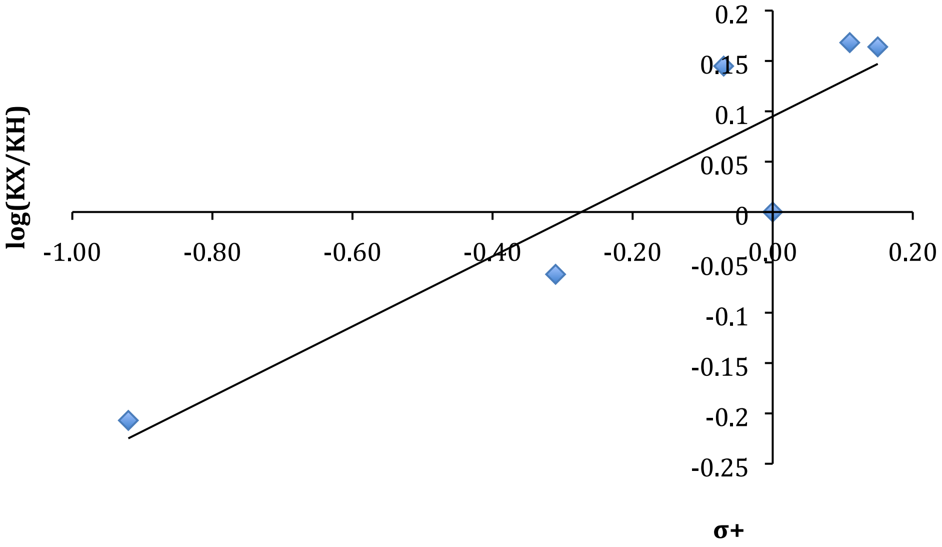

Figure S2. Hammett plot (σ^+) for four-component assembly with para substituted **2-PA**.

NMR and Mass Spectra:

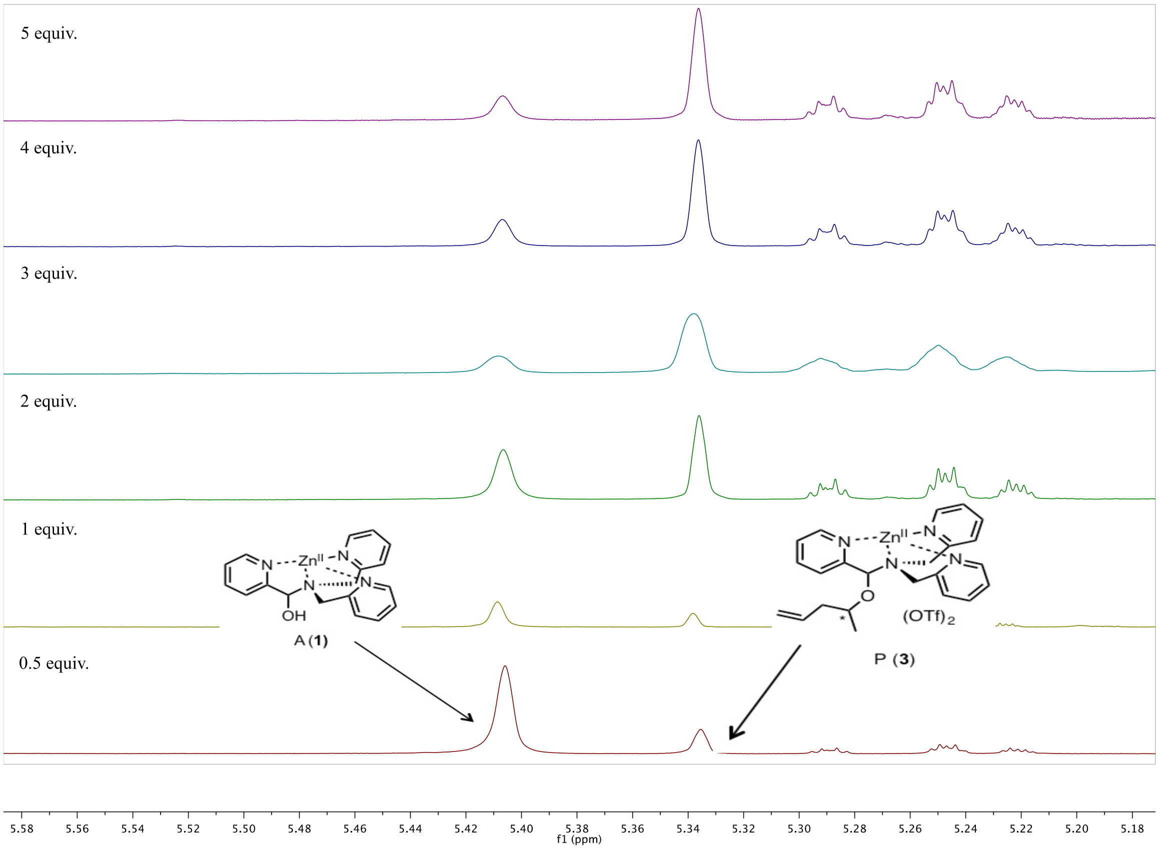

Figure S4. ^{1}H NMR of multi-component assembly varying the concentration of alcohol from 17.5 mM (0.5 equiv.) to 210 mM (6 equiv.). For alcohol, 4-penten-2-ol was chosen for all kinetic studies.

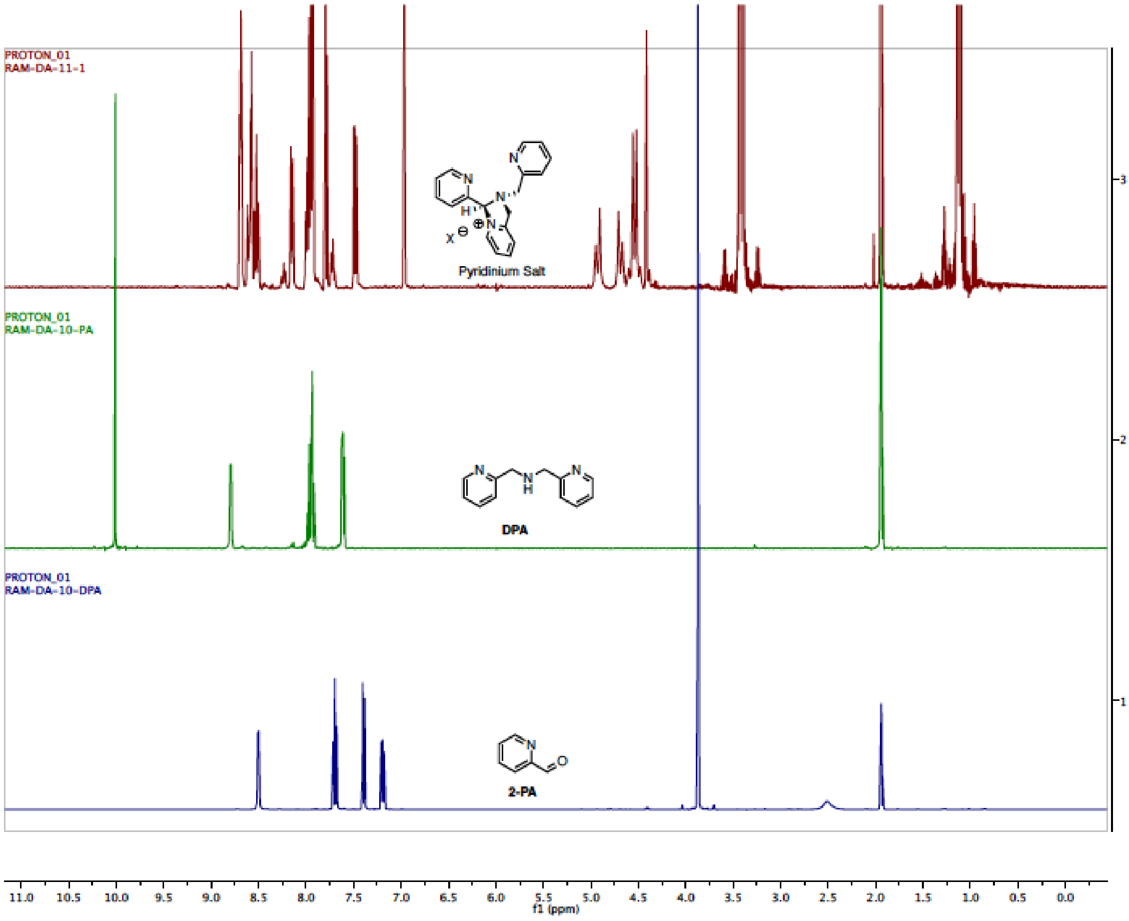

Figure S5. ^{1}H NMR of pyridinium salt (**5**) formed from **2-PA**(1.0 equiv.), **DPA** (1.2 equiv.), and BF_3-OEt_2 (1.2 equiv.) (top), ^{1}H NMR of **DPA** (middle) and ^{1}H NMR of **2-PA** (bottom). All nmrs recorded in CD_3CN.

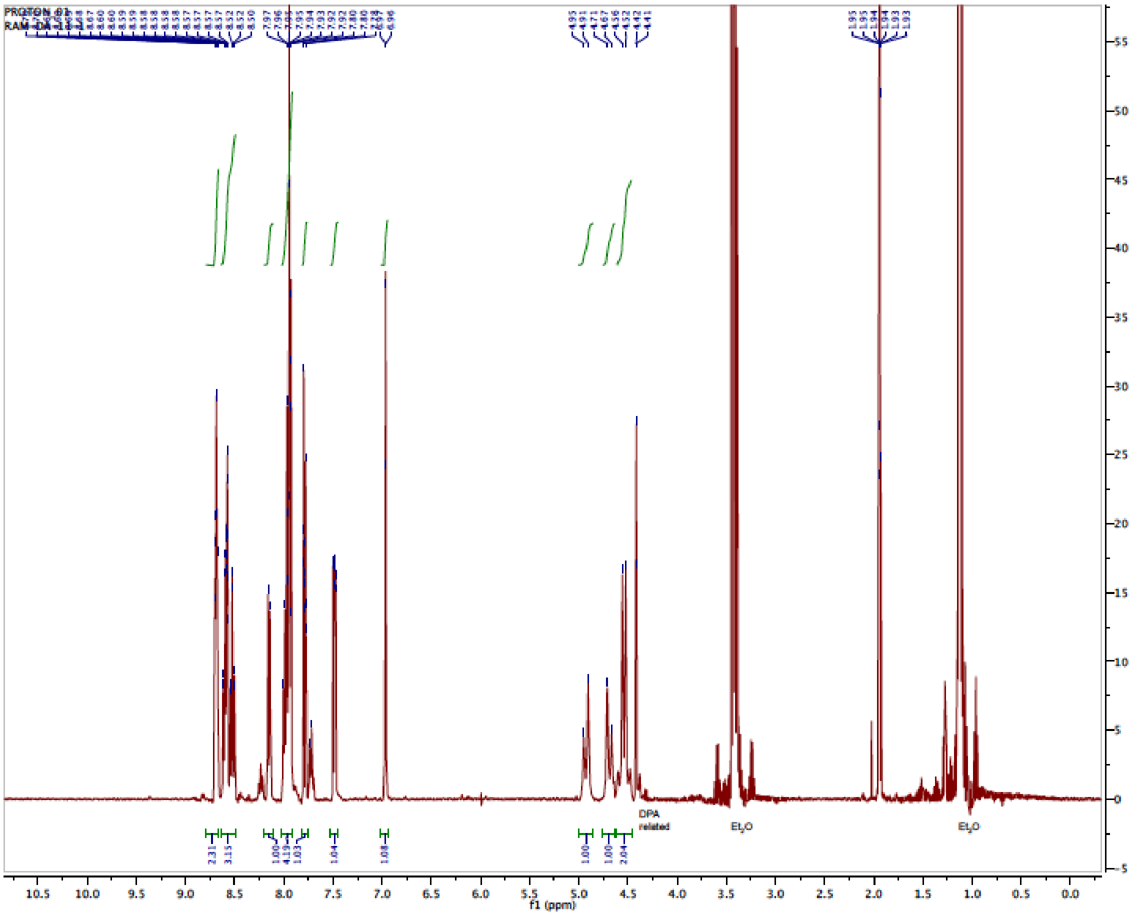

Figure S6. ^{1}H NMR of pyridinium salt (**5**) formed from **2-PA**(1.0 equiv.), **DPA** (1.2 equiv.), and BF_3-OEt_2 (1.2 equiv.) in CD_3CN.

Peaks corresponding to pyridinium salt (**5**): ^{1}H NMR (400 MHz, CD_3CN) δ 8.72–8.66 (m, 2H), 8.62–8.50 (m, 3H), 8.15 (d, J = 8.0 Hz, 1H), 8.00–7.91 (m, 4H), 7.79 (d, J = 8.0 Hz, 1H), 7.48 (m, 1H), 6.97 (s, 1H), 4.93 (d, J = 16.8 Hz, 1H), 4.69 (d, J = 16.8 Hz, 1H), 4.58 (d, J = 16.8 Hz, 1H), 4.50 (d, J = 16.8 Hz, 1H).

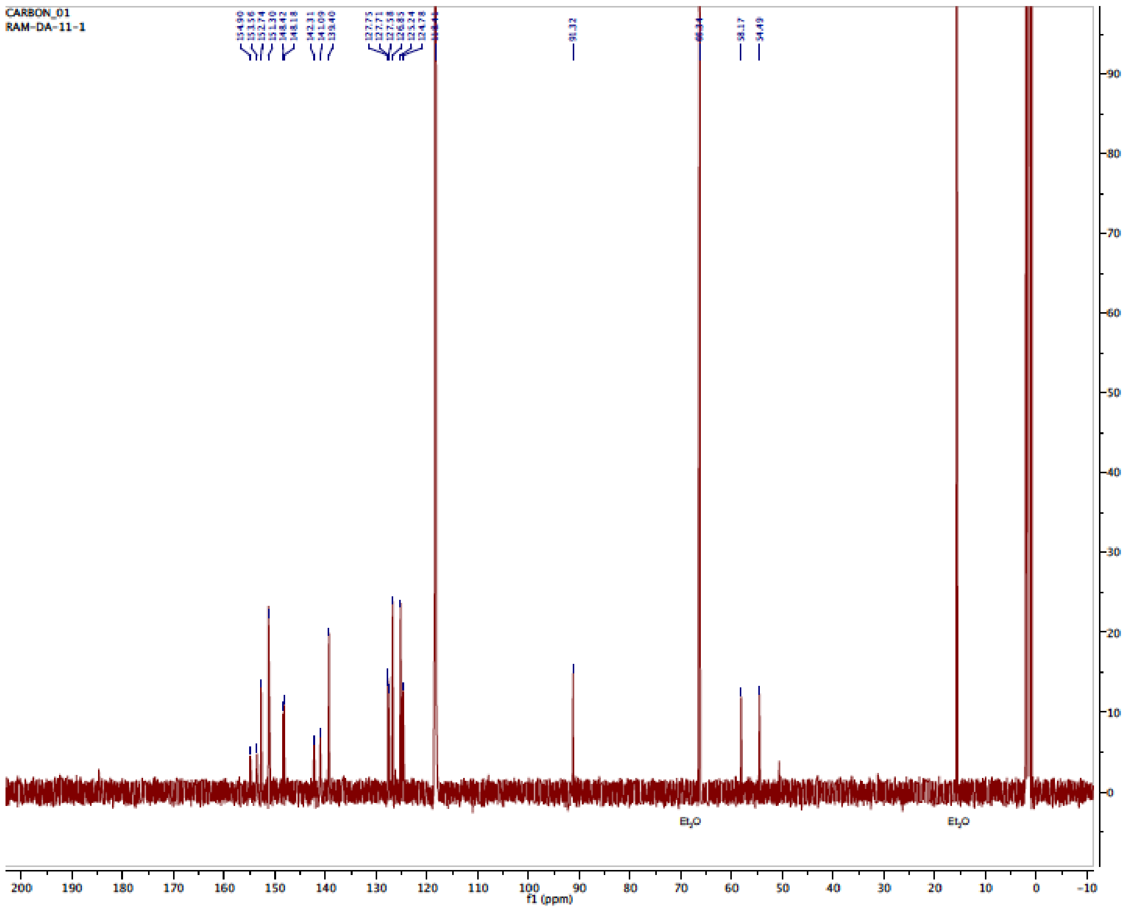

Figure S7. ^{13}C NMR of pyridinium salt (**5**) formed from **2-PA**(1.0 equiv.), **DPA** (1.2 equiv.), and BF_3-OEt_2 (1.2 equiv.) in CD_3CN.

Peaks corresponding to pyridinium salt (**5**): ^{13}C NMR (100 MHz, CD_3CN) δ 154.9, 153.6, 152.7, 151.3, 148.4, 148.2, 142.3, 141.1, 139.4, 127.8, 127.7, 127.6, 126.9, 125.2, 124.5, 91.3, 58.2, 54.5.

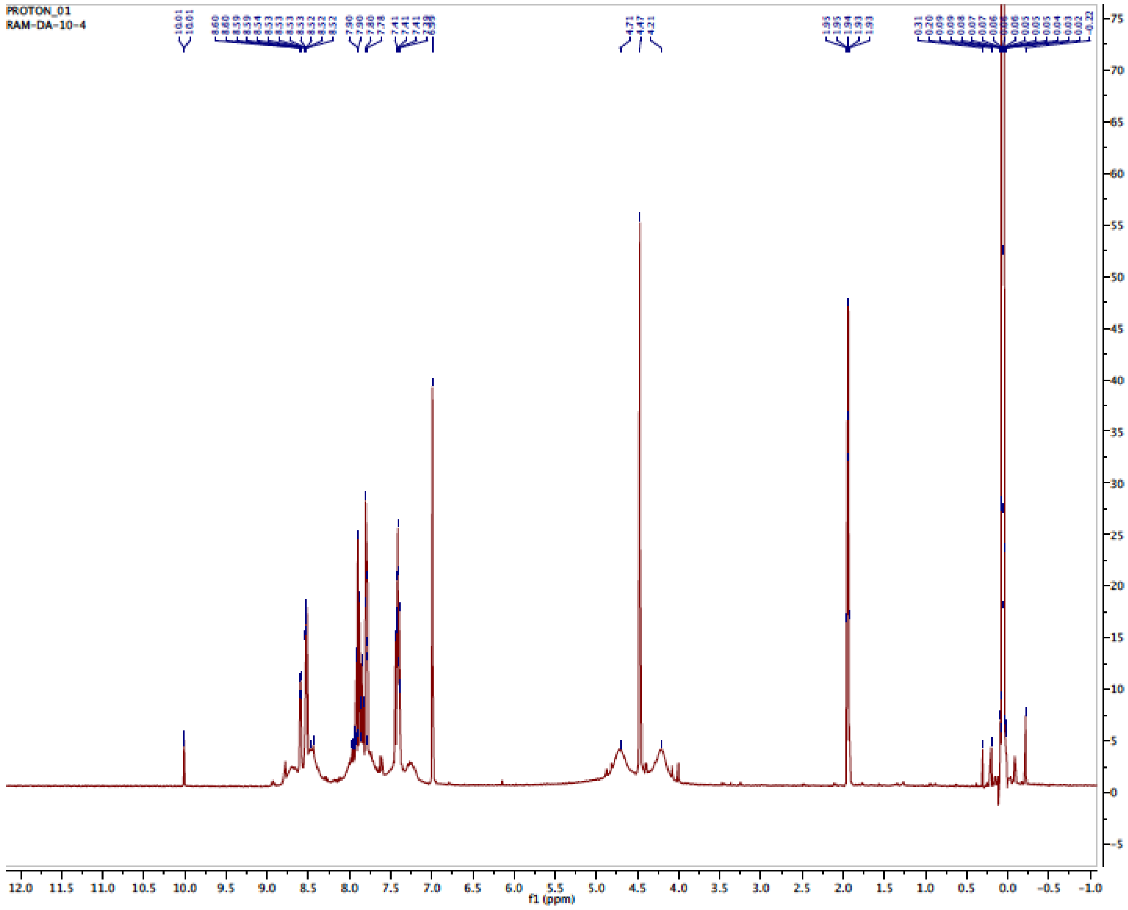

Figure S8. ^{1}H NMR of pyridinium salt (**5**) formed from **2-PA** (1.0 equiv.), **DPA** (1.2 equiv.) and TMS-OTf (1.2 equiv.) in CD_3CN.

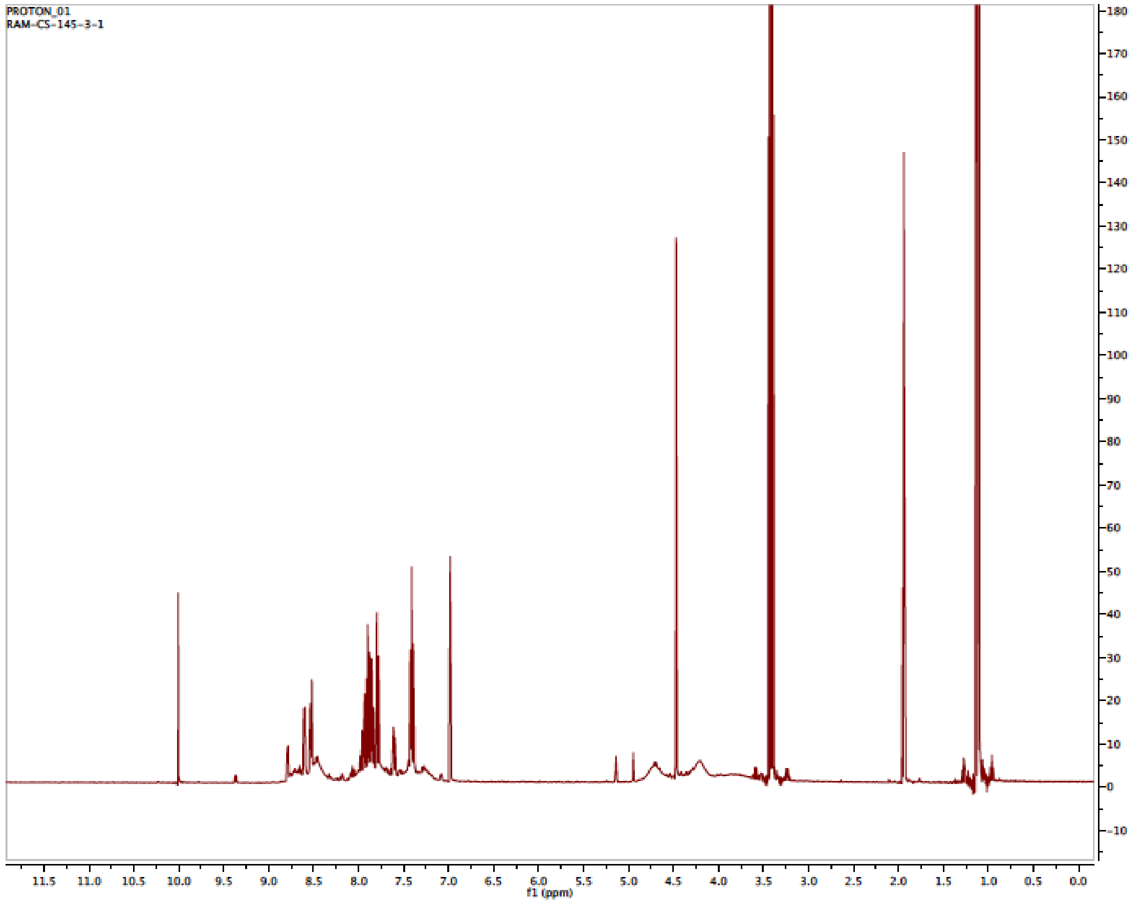

Figure S9. ^{1}H NMR of pyridinium salt (**5**) formed from **2-PA** (1.0 equiv.), **DPA** (1.0 equiv.) and BF_3-OEt_2 (1.0 equiv.) in CD_3CN.

**Data File**	MSF14-2504(RAM-CS-157-C)_hrESIpos3.d	**Sample Name**	2504	**Comment**	RAM-CS-157-C
**Position**	P1-D4	**Instrument Name**	Instrument 1	**User Name**	
**Acq Method**	pos.m	**Acquired Time**	5/8/2014 6:15:48 PM	**DA Method**	Ian.m

MS Zoomed Spectrum

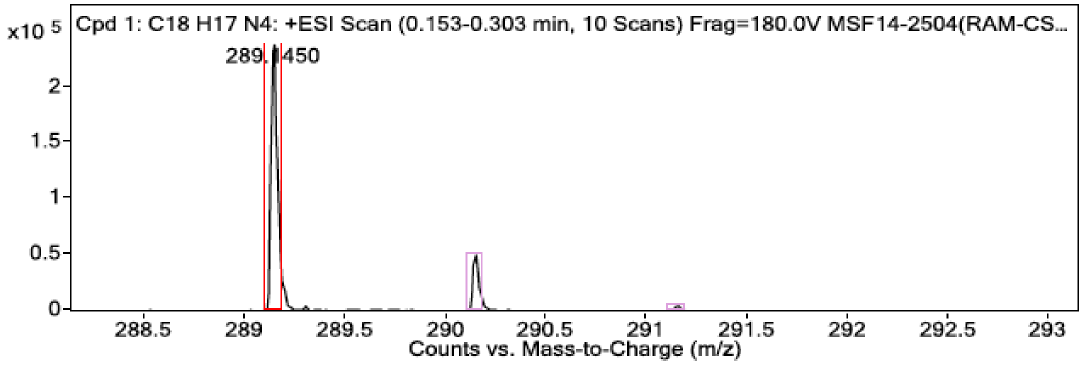

MS Spectrum Peak List

Obs. m/z	Calc. m/z	Charge	Abund	Formula	Ion/Isotope	Tgt Mass Error (ppm)
289.14500	289.14480	1	244633.53	C18H17N4	M+	-0.69

— End Of Report —

Figure S10. Mass spectrum of pyridinium salt (**5**) generated from **2-PA**, **DPA** and BF_3-OEt_2. HRMS calcd for $C_{18}H_{17}N_4^+$ (M^+) 289.1448. Found 289.1450.

**Data File**	MSF14-2503(RAM-CS-157-A)_hrESIpos1.d	**Sample Name**	2503	**Comment**	RAM-CS-157-A
**Position**	P1-D3	**Instrument Name**	Instrument 1	**User Name**	
**Acq Method**	pos.m	**Acquired Time**	5/8/2014 5:59:58 PM	**DA Method**	Ian.m

MS Zoomed Spectrum

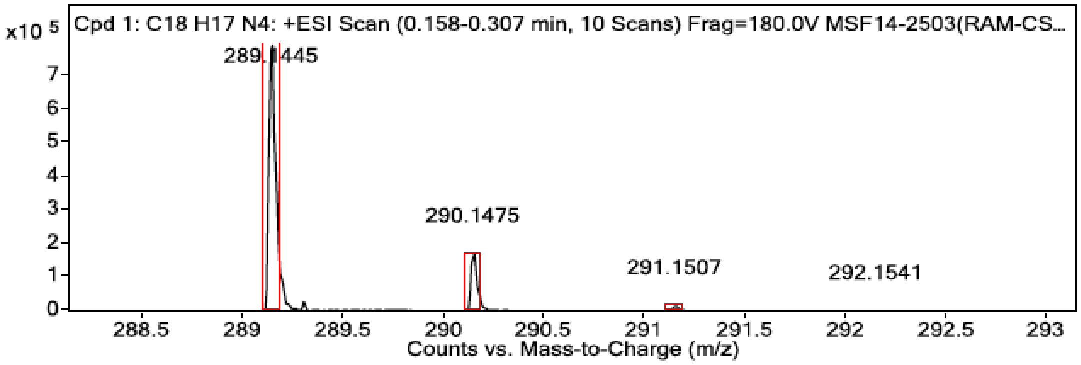

MS Spectrum Peak List

Obs. m/z	Calc. m/z	Charge	Abund	Formula	Ion/Isotope	Tgt Mass Error (ppm)
289.14450	289.14480	1	816216.9	C18H17N4	M+	1.01
290.14750	290.14770	1	171349.73	C18H17N4	M+	0.92
291.15070	291.15060	1	16915.33	C18H17N4	M+	-0.31
292.15410	292.15350	1	1405.73	C18H17N4	M+	-2.12
343.15280			855700.24			

--- End Of Report ---

Figure S11. Mass spectrum of pyridinium salt generated from **2-PA**, **DPA** and TMS-OTf. HRMS calcd for $C_{18}H_{17}N_4^+$ (M^+) 289.1448. Found 289.1445.
